# Supplementary material for: Metabolomics-guided discovery of cytochrome P450s involved in pseudotropine-dependent biosynthesis of modified tropane alkaloids
Source: Nat Commun. 2022 Jul 2;13:3832. doi: 10.1038/s41467-022-31653-1 (PMC9250511; doi:10.1038/s41467-022-31653-1)
Supplement: Supplementary file 1 — Supplementary Information [file 41467_2022_31653_MOESM1_ESM.pdf]

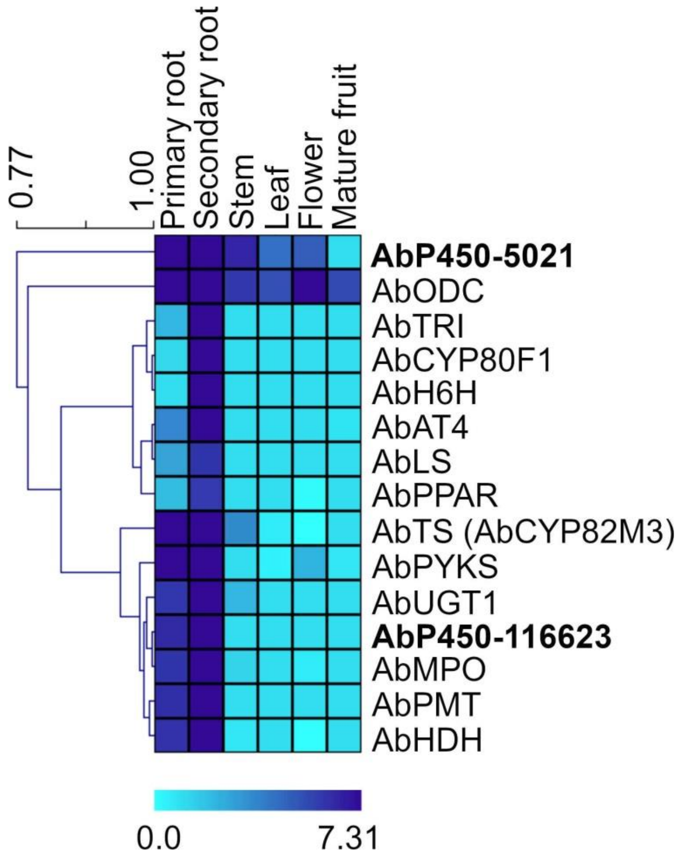

Supplementary Figure 1. Heat map of expression data for *AbP450-5021*, *AbP450-116623* and the known scopolamine biosynthesis pathway genes in *Atropa belladonna*. Hierarchical clustering was generated based on average linkage of Pearson correlation coefficients of log_2-transformed FPKM expression values from the MPGR website (http://medicinalplantgenomics.msu.edu/). The color scale depicts transcript abundance expressed as log_2-transformed FPKM. Source data are provided as a Source Data file. AbP450-5021, functionally characterized in this study; AbP450-116623, functionally characterized in this study; scopolamine biosynthesis: AbODC, ornithine decarboxylase; AbTRI, tropinone reductase I; AbCYP80F1, littorine mutase; AbH6H, hyoscyamine 6β-hydroxylase; AbAT4, aromatic amino acid aminotransferase 4; AbLS, littorine synthase; AbPPAR, phenylpyruvic acid reductase; AbTS, tropinone synthase; AbPYKS, type III polyketide synthase; AbUGT1, phenyllactate UDP-glycosyltransferase; AbMPO, *N*-methylputrescine oxidase; AbPMT, putrescine *N*-methyltransferase; AbHDH, hyoscyamine dehydrogenase

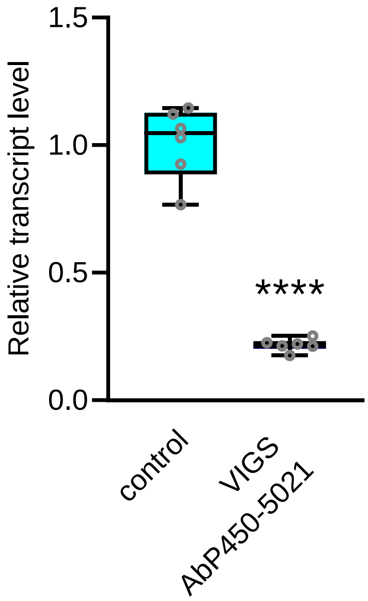

Supplementary Figure 2. Silencing of *AbP450-5021* in *Atropa belladonna* VIGS lines. VIGS decreased the target gene transcript level on average by 79% in *AbP450-5021* VIGS lines compared to the respective group of control lines (empty vector). The relative *AbP450-5021* transcript level is shown for six individual *AbP450-5021* VIGS lines compared to six control lines. The plants are biological replicates (*N*=6), selected based on their calystegine A5 and A3 levels closest to the median values for the given genotypes. Data are presented as mean of three technical replicates for each individual line. Asterisks indicate significant differences (**** $p < 0.00005$) as determined by Student's *t* test (unpaired, two-tailed, Welch correction). For each box plot, the lower and upper bounds of the box represent the first and third quartiles, the line indicates the median value, and the whiskers extend to the minimum and maximum data points. Source data are provided as a Source Data file.

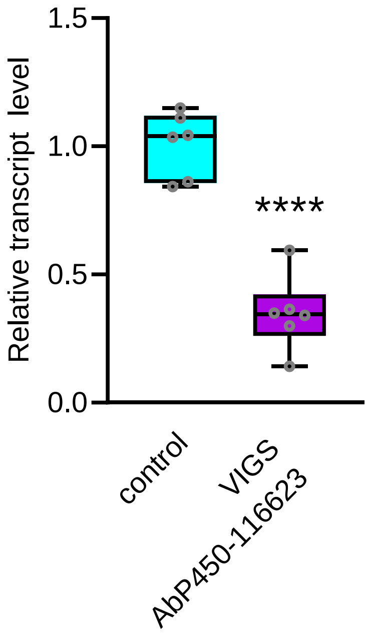

Supplementary Figure 3. Silencing of *AbP450-116623* in *Atropa belladonna* VIGS lines. VIGS decreased the target gene level on average by 65% in *AbP450-116623* VIGS lines compared to the respective group of control lines (empty vector). The relative *AbP450-116623* transcript level is shown for six individual *AbP450-116623* VIGS lines compared to six control lines. The plants are biological replicates, (N=6) selected based on their calystegine A5 and A3 levels closest to the median values for the given genotypes. Data are presented as mean of three technical replicates for each individual line. Asterisks indicate significant differences (**** $p < 0.00005$) as determined by Student's t test (unpaired, two-tailed, Welch correction). For each box plot, the lower and upper bounds of the box represent the first and third quartiles, the line indicates the median value, and the whiskers extend to the minimum and maximum data points. Source data are provided as a Source Data file.

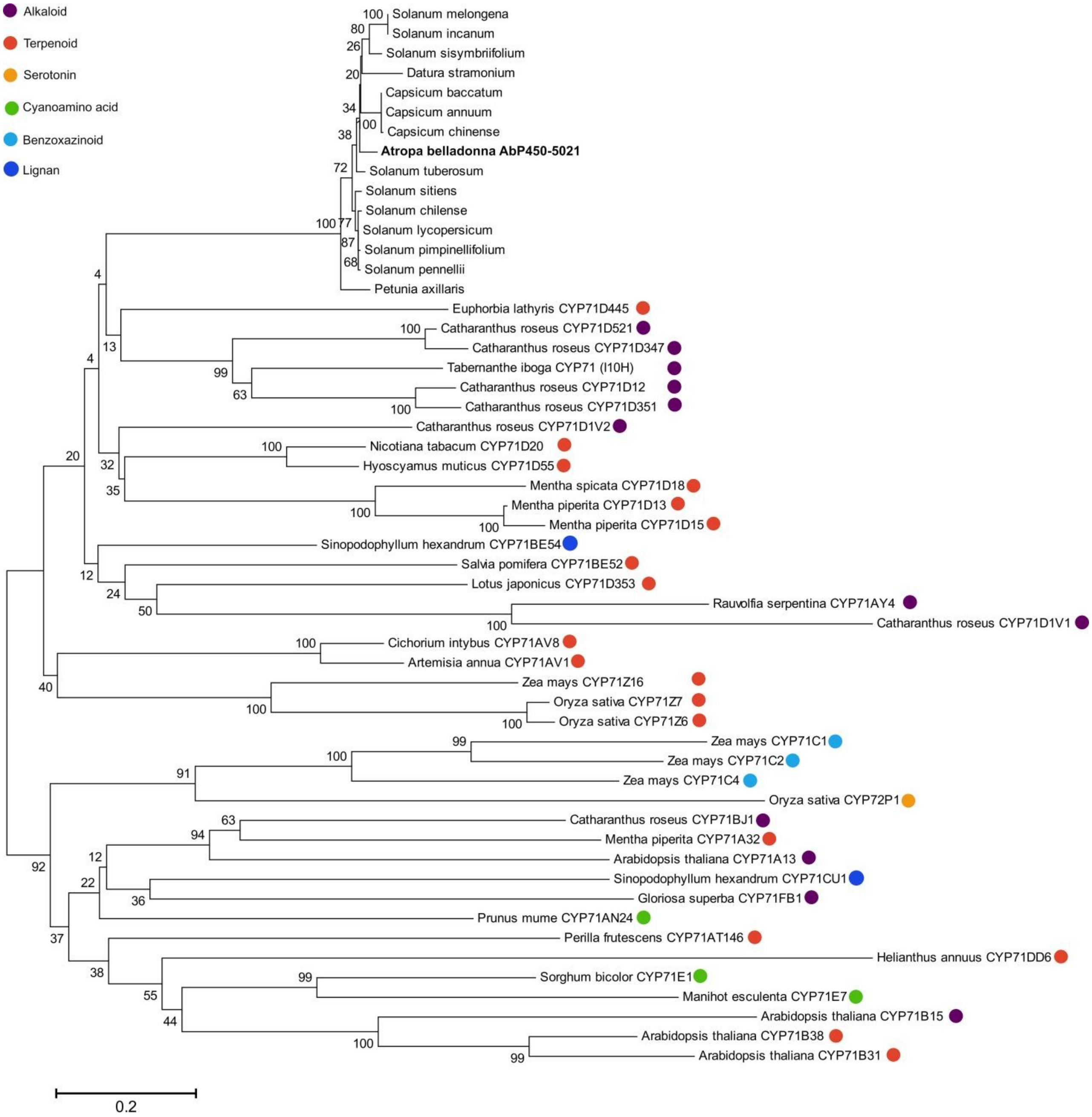

Supplementary Figure 4. Phylogenetic relationship of AbP450-5021, AbP450-5021 homologs in the Solanaceae family, and functionally characterized members of the CYP71 family. Each of the predicted AbP450-5021 homologs in the Solanaceae family represents the most closely related protein to AbP450-5021 in a given species (sequence identities range from 89-93%). In the tree, *Atropa belladonna* AbP450-5021 (this study) is highlighted. Dots indicate functionally characterized CYP71 enzymes in specialized metabolism. Protein sequences were aligned with ClustalW, and the neighbor-joining tree was constructed with MEGA 6.06. The complete deletion option was used, and bootstrap values were determined from 1,000 replicates. Evolutionary distances were computed using the Poisson

correction method. The scale bar represents 0.2 amino acid substitutions per site. Sequences included in this analysis are provided as a Source Data file.

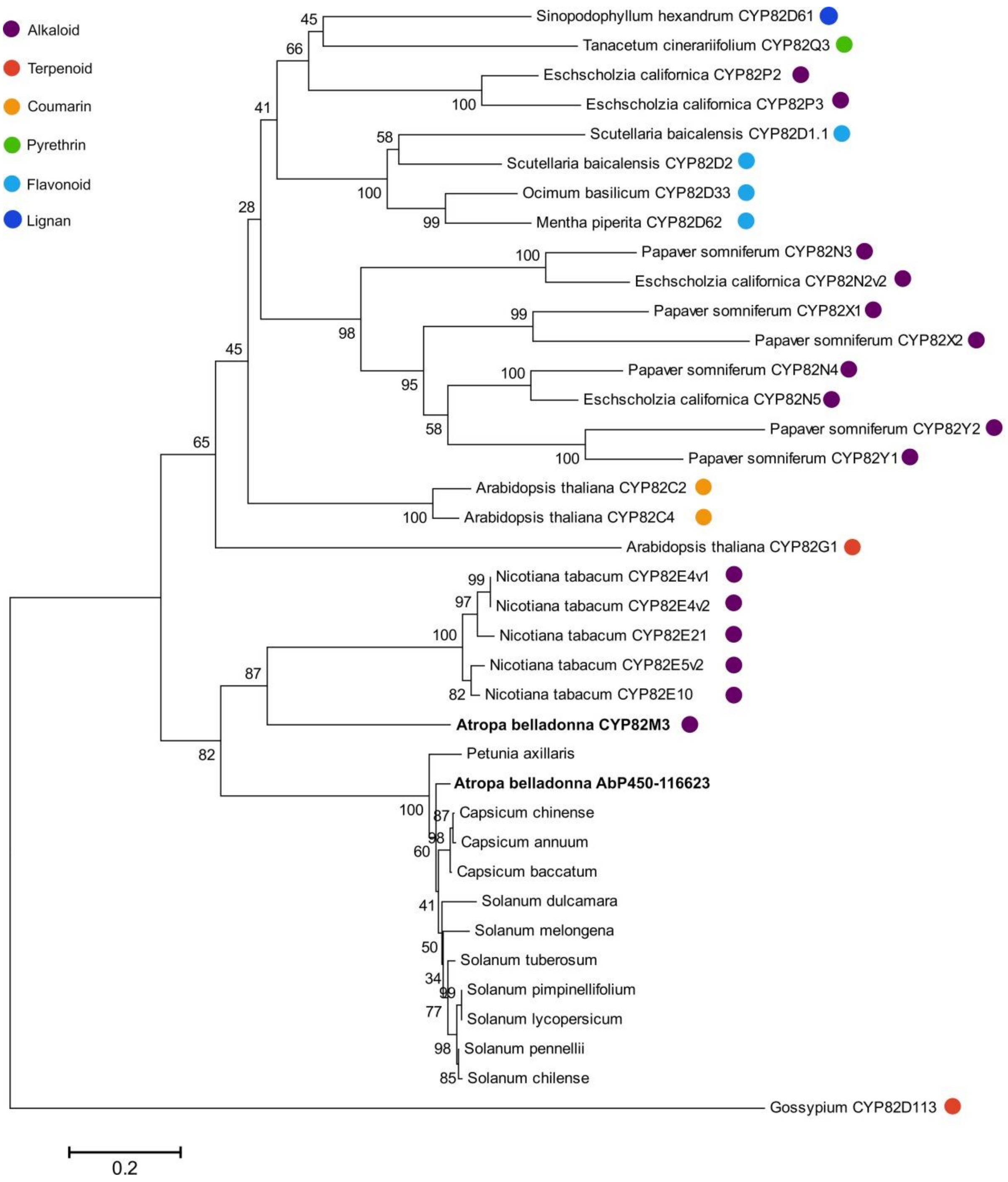

Supplementary Figure 5. Phylogenetic relationship of AbP450-116623, AbP450-116623 homologs in the Solanaceae family, and functionally characterized members of the CYP82 family. Each of the predicted AbP450-116623 homologs in the Solanaceae family represents the most closely related protein in a given species (sequence identities range from 85-91%). In the tree, *Atropa belladonna* AbP450-116623 (this study) and CYP82M3 (tropinone synthase) are highlighted. Dots indicate functionally characterized enzymes in specialized

metabolism. Most of the characterized CYP82 enzymes catalyze hydroxylation reactions. Exceptions are the isofunctional *Nicotiana tabacum N*-demethylases CYP82E4v1, CYP82E4v1, CYP82E21, CYP825v2, and CYP82E10, *Papaver somniferum* CYP82Y2 epimerase, CYP82Q3 catalyzing desaturation, and CYP82G1 catalyzing oxidative degradation. Protein sequences were aligned with ClustalW, and the neighbor-joining tree was constructed with MEGA 6.06. The complete deletion option was used, and bootstrap values were determined from 1,000 replicates. Evolutionary distances were computed using the Poisson correction method. The scale bar represents 0.2 amino acid substitutions per site. Sequences included in this analysis are provided as a Source Data file.

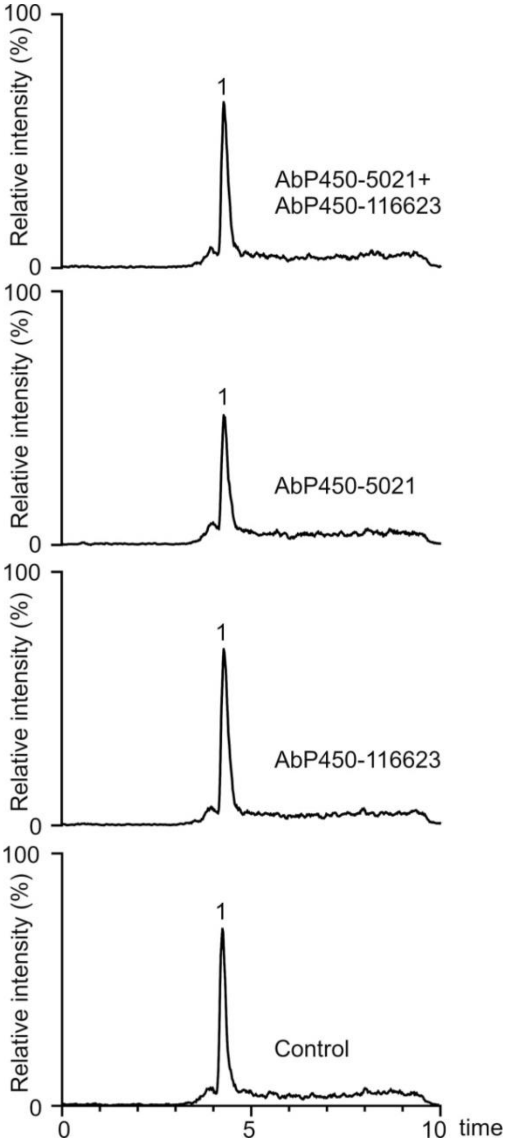

Supplementary Figure 6. *N. benthamiana* transient assays with acetyl pseudotropine. *N. benthamiana* leaves producing AbP450-5021, AbP450-116623 and AbP450-5021+AbP450-116623 were assayed with acetyl pseudotropine for *N*-demethylase and ring-hydroxylase activity (Fig. 3, Supplementary Tables 3 and 4). Control experiments were conducted with infiltrated leaves that did not produce *A. belladonna* cytochrome P450s. Leaf metabolite extracts were analyzed by UHPLC/MS using a 10-min gradient elution method. Each condition was tested in triplicate, and the experiments were replicated twice. Representative chromatograms are shown. Each chromatogram represents a combined extracted ion chromatogram for the exact masses of acetyl pseudotropine (1), acetyl norpseudotropine, and acetyl hydroxynorpseudotropine. No conversion of acetyl pseudotropine to acetyl norpseudotropine and acetyl hydroxynorpseudotropine was detected. Note the absolute vertical scale (Y-axis) is identical for the chromatograms shown.

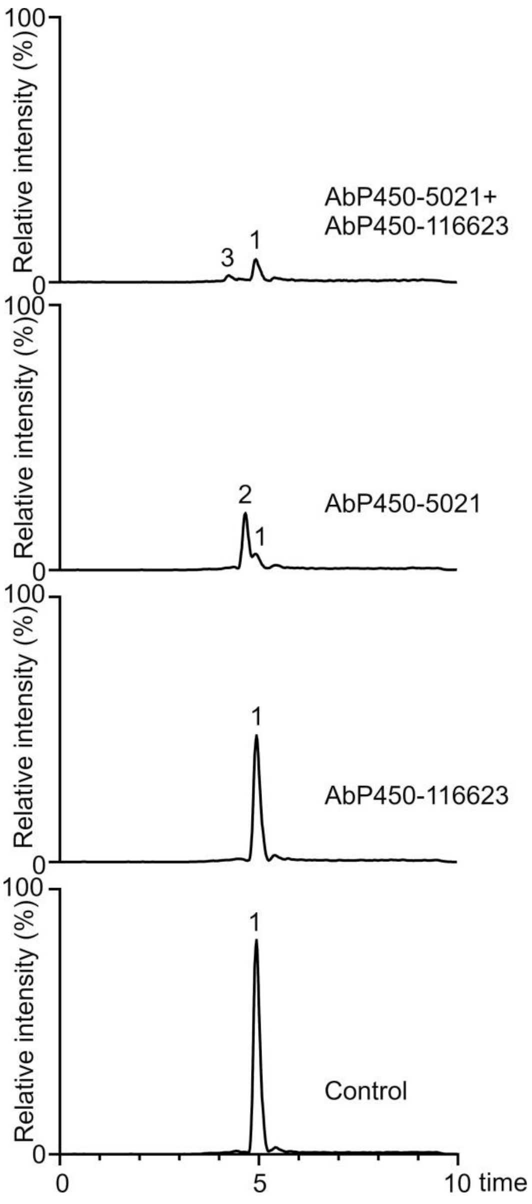

Supplementary Figure 7. *N. benthamiana* transient assays with propionyl pseudotropine. *N. benthamiana* leaves producing AbP450-5021, AbP450-116623 or AbP450-5021+AbP450-116623 were assayed with propionyl pseudotropine for *N*-demethylase and ring-hydroxylase activity (Fig. 3, Supplementary Tables 3 and 4). Control experiments were conducted with infiltrated leaves that did not produce *A. belladonna* cytochrome P450s. Leaf metabolite extracts were analyzed by UHPLC/MS using a 10-min gradient elution method. Each condition was tested in triplicate, and the experiments were replicated twice. Representative chromatograms are shown. Each chromatogram represents a combined extracted ion chromatogram for the exact masses of propionyl pseudotropine (1), propionyl norpseudotropine (2), and propionyl hydroxynorpseudotropine (3). Note the absolute vertical scale (Y-axis) is identical for the chromatograms shown.

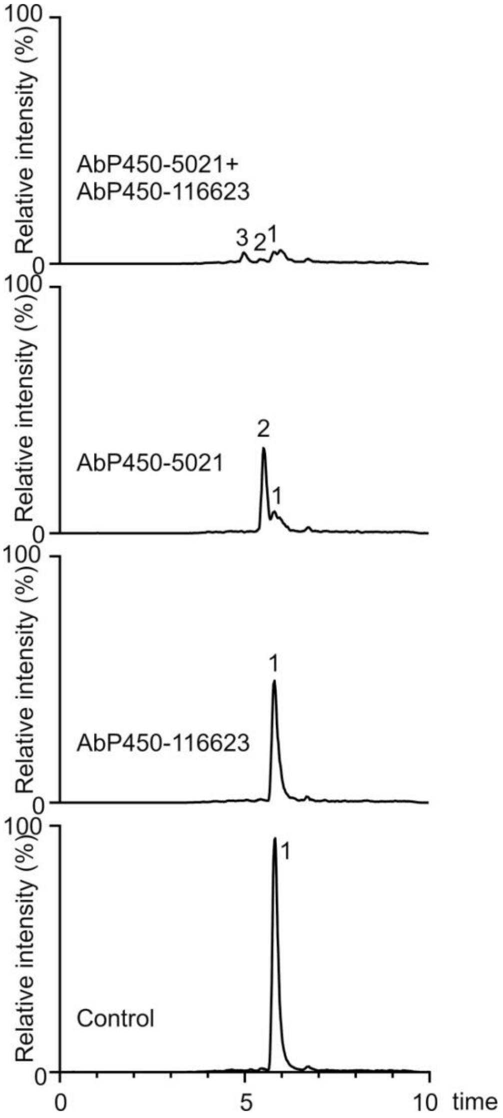

Supplementary Figure 8. *N. benthamiana* transient assays with isobutyryl pseudotropine. *N. benthamiana* leaves producing AbP450-5021, AbP450-116623 or AbP450-5021+AbP450-116623 were assayed with isobutyryl pseudotropine for *N*-demethylase and ring-hydroxylase activity (Fig. 3, Supplementary Tables 3 and 4). Control experiments were conducted with infiltrated leaves that did not produce *A. belladonna* cytochrome P450s. Leaf metabolite extracts were analyzed by UHPLC/MS using a 10-min gradient elution method. Each condition was tested in triplicate, and the experiments were replicated twice. Representative chromatograms are shown. Each chromatogram represents a combined extracted ion chromatogram for the exact masses of isobutyryl pseudotropine (1), isobutyryl norpseudotropine (2), and isobutyryl hydroxynorpseudotropine (3). Note the absolute vertical scale (Y-axis) is identical for the chromatograms shown.

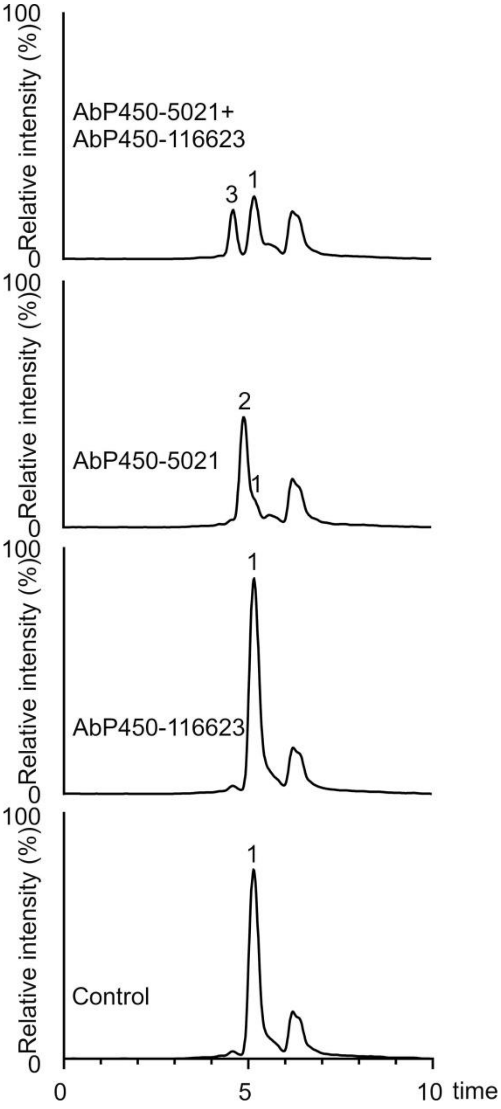

Supplementary Figure 9. *N. benthamiana* transient assays with isovaleryl pseudotropine. *N. benthamiana* leaves producing AbP450-5021, AbP450-116623 or AbP450-5021+AbP450-116623 were assayed with isovaleryl pseudotropine for *N*-demethylase and ring-hydroxylase activity (Fig. 3, Supplementary Tables 3 and 4). Control experiments were conducted with infiltrated leaves that did not produce *A. belladonna* cytochrome P450s. Leaf metabolite extracts were analyzed by UHPLC/MS using a 10-min gradient elution method. Each condition was tested in triplicate, and the experiments were replicated twice. Representative chromatograms are shown. Each chromatogram represents a combined extracted ion chromatogram for the exact masses of isovaleryl pseudotropine (1), isovaleryl norpseudotropine (2), and isovaleryl hydroxynorpseudotropine (3). Note the absolute vertical scale (Y-axis) is identical for the chromatograms shown.

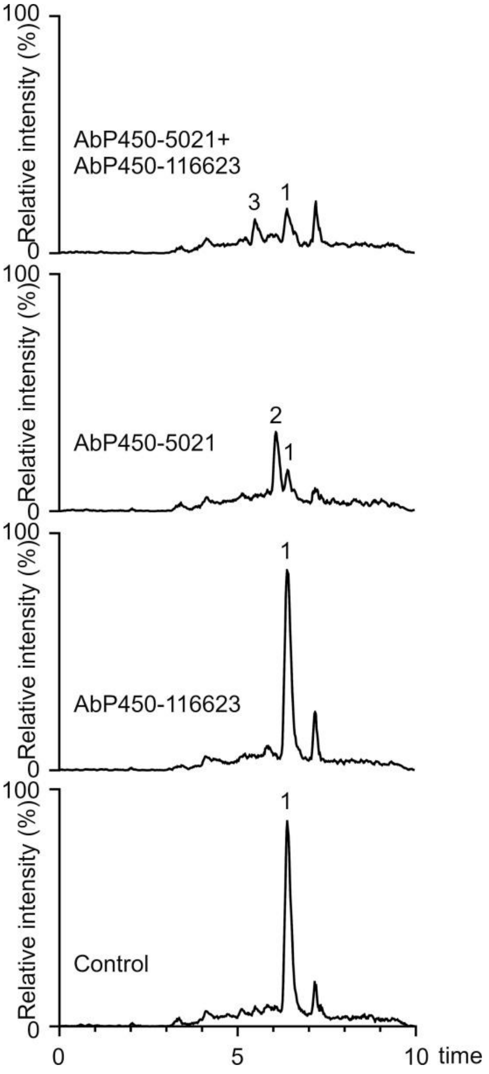

Supplementary Figure 10. *N. benthamiana* transient assays with senecioyl pseudotropine. *N. benthamiana* leaves producing AbP450-5021, AbP450-116623 or AbP450-5021+AbP450-116623 were assayed with senecioyl pseudotropine for *N*-demethylase and ring-hydroxylase activity (Fig. 3, Supplementary Tables 3 and 4). Control experiments were conducted with infiltrated leaves that did not produce *A. belladonna* cytochrome P450s. Leaf metabolite extracts were analyzed by UHPLC/MS using a 10-min gradient elution method. Each condition was tested in triplicate, and the experiments were replicated twice. Representative chromatograms are shown. Each chromatogram represents a combined extracted ion chromatogram for the exact masses of senecioyl pseudotropine (1), senecioyl norpseudotropine (2), and senecioyl hydroxynorpseudotropine (3). Note the absolute vertical scale (Y-axis) is identical for the chromatograms shown.
