# Supplementary material for: Metabolomics-guided discovery of cytochrome P450s involved in pseudotropine-dependent biosynthesis of modified tropane alkaloids
Source: Nat Commun. 2022 Jul 2;13:3832. doi: 10.1038/s41467-022-31653-1 (PMC9250511; doi:10.1038/s41467-022-31653-1)
Supplement: Supplementary file 3 — Description of Additional Supplementary Files [file 41467_2022_31653_MOESM3_ESM.pdf]

Description of Additional Supplementary Files

File Name: Supplementary Data 1
Description: Alkaloids in Atropa belladonna root.

File Name: Supplementary Data 2
Description: Tropine, littorine, hyoscyamine and scopolamine levels in Atropa roots.

File Name: Supplementary Data 3
Description: UHPLC-MS/MS analysis of substrates and enzymatic products in transient assays.

File Name: Supplementary Data 4
Description: Transient assays in Nicotiana benthamiana.

File Name: Supplementary Data 5
Description: Oligonucleotide primers.

File Name: Supplementary Data 6
Description: NMR data.
