# Supplementary material for: Metabolomics-guided discovery of cytochrome P450s involved in pseudotropine-dependent biosynthesis of modified tropane alkaloids
Source: Nat Commun. 2022 Jul 2;13:3832. doi: 10.1038/s41467-022-31653-1 (PMC9250511; doi:10.1038/s41467-022-31653-1)
Supplement: Supplementary file 9 — Supplementary Data 6 [file 41467_2022_31653_MOESM9_ESM.pdf]

O-Acyl pseudotropine

A. 3-Acetyl pseudotropine

NMR chemical shifts values for 3-Acetyl pseudotropine

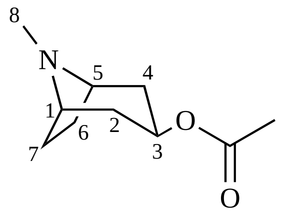

8-methyl-8-azabicyclo[3.2.1]octan-3β-yl acetate
(3-Acetyl pseudotropine)

Chemical Formula: $C_{10}H_{17}NO_2$
Experimental *m/z*: 184.1332
Theoretical *m/z* of $[M+H]^+$: 184.13321
InChI Key: MDIDMOWWLBGYPG-ILWJIGKKSA-N
SMILES:
[H][C@]1(OC(C)=O)C[C@@H]2CC[C@@H](N2C)C1
NMR (500 MHz, D_2O) ~2 mg

**Carbon # (group)**	**^{1}H (ppm)**	**^{13}C (ppm) From HMBC and HSQC**
**1,5** (CH)	3.81 (p, *J* = 6.2 Hz, 2H)	63.15
**2,4** (CH_2)	Axial 1.80 (m, *J* = 14.4, 11.0, 6.2 Hz, 2H)<br>Equatorial 2.16 (dt, *J* = 14.4, 6.2 Hz, 2H)	34.59
**3** (CH)<br>- 1 (CO)<br>- 2 (CH_3)	5.00 (tt, *J* = 11.0, 6.2 Hz, 1H)<br>-<br>1.90 (s, 3H)	64.30<br>174.18<br>20.48
**6,7** (CH_2)	1.95 (dd, *J* = 6.2, 14.2 Hz, 2H), 2.17 (m, *J* = 14.2 Hz, 2H)	23.48
**8** N(CH_3)	2.35 (s, 3H)	38.03

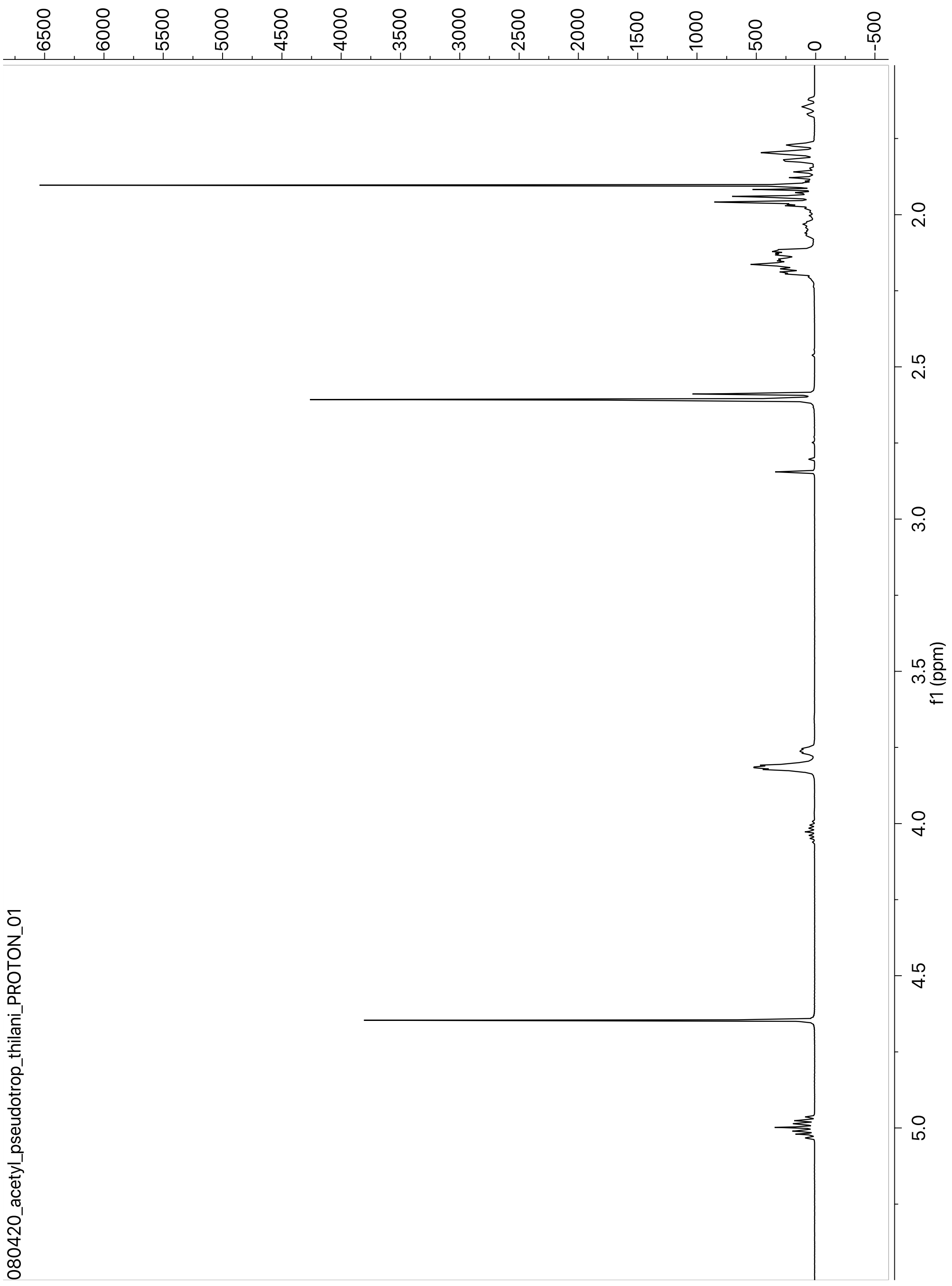

^{1}H NMR spectrum for 3-acetyl pseudotropine

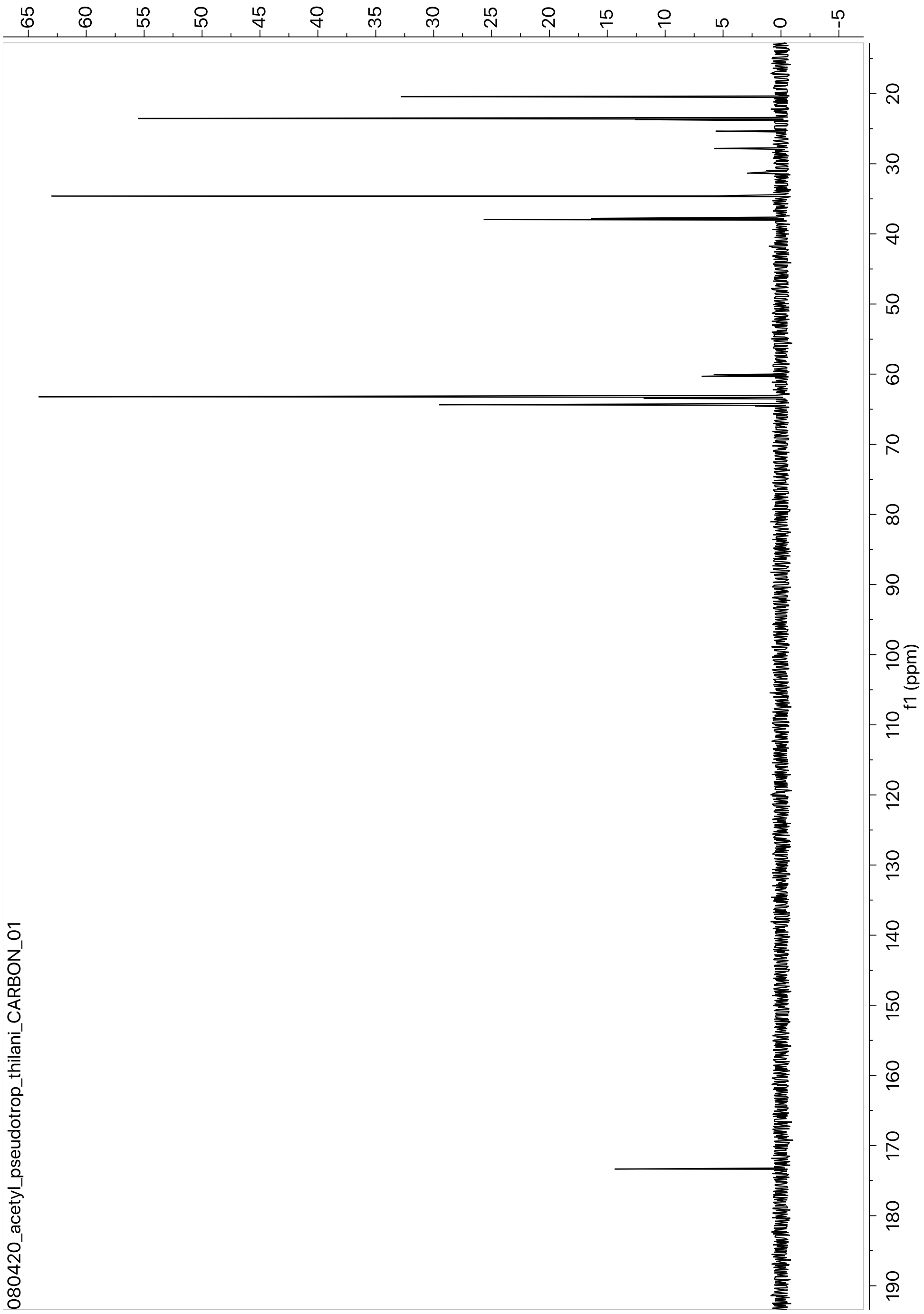

^{13}C NMR spectrum for 3-acetyl pseudotropine

B. 3-Propionyl pseudotropine

NMR chemical shifts values for 3-propionyl pseudotropine

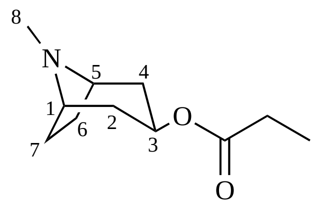	8-methyl-8-azabicyclo[3.2.1]octan-3β-yl propionate<br>(3-Propionyl pseudotropine)<br><br>Chemical Formula: $C_{11}H_{19}NO_2$<br>Experimental *m/z* of $[M+H]^+$: 198.1494<br>Theoretical *m/z* of $[M+H]^+$: 198.14886<br>InChI Key: NUFSBXOAMBFLRJ-ILWJIGKKSA-N<br>SMILES:<br>[H][C@]1(OC(CC)=O)C[C@@H]2CC[C@@H](N2C)C1<br>NMR (500 MHz, $CDCl_3$) ~2 mg	
**Carbon #<br>(group)**	**^{1}H (ppm)**	**^{13}C (ppm)**
**1,5** (CH)	3.22 (p, *J* = 5.0 Hz, 2H)	60.29
**2,4** (CH_2)	Axial 1.76 (t, *J* = 5.0 Hz, 2H)<br>Equatorial 1.83 (dt, *J* = 16.5, 5.0 Hz, 2H)	35.45
**3** (CH)<br>- 1 (CO)<br>- 2 (CH_2)<br>- 2 (CH_3)	4.99 (tt, *J* = 5.0 Hz, 1H)<br>-<br>2.27 (q, *J* = 7.6 Hz, 2H)<br>1.10 (t, *J* = 7.6 Hz, 3H)	66.81<br>174.18<br>27.85<br>9.17
**6,7** (CH_2)	1.66 (dd, *J* = 5.0, 13.5 Hz, 2H), 2.04 (m, *J* = 13.5 Hz, 2H)	26.50
**8** N(CH_3)	2.35 (s, 3H)	38.70

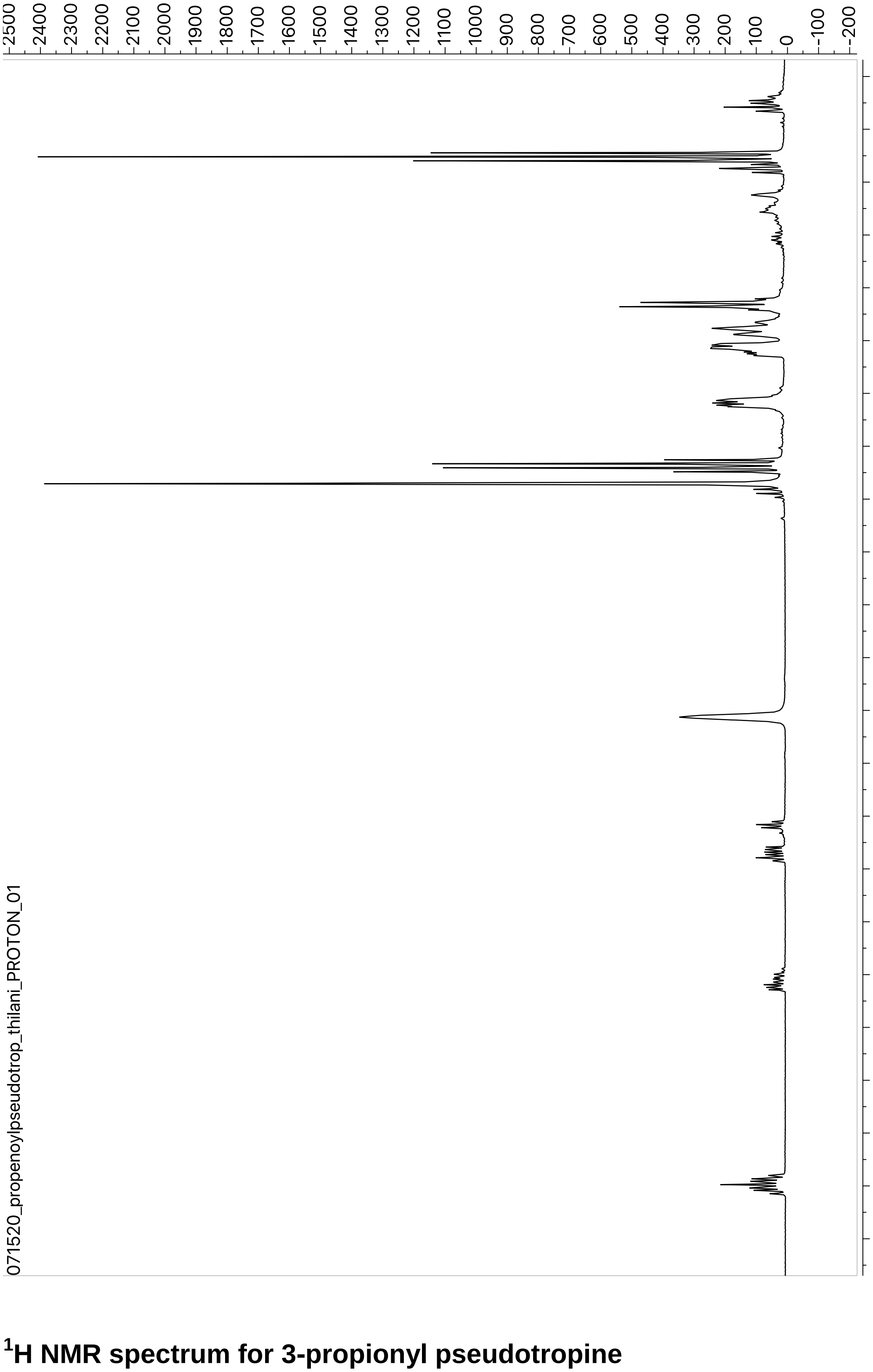

^{1}H NMR spectrum for 3-propionyl pseudotropine

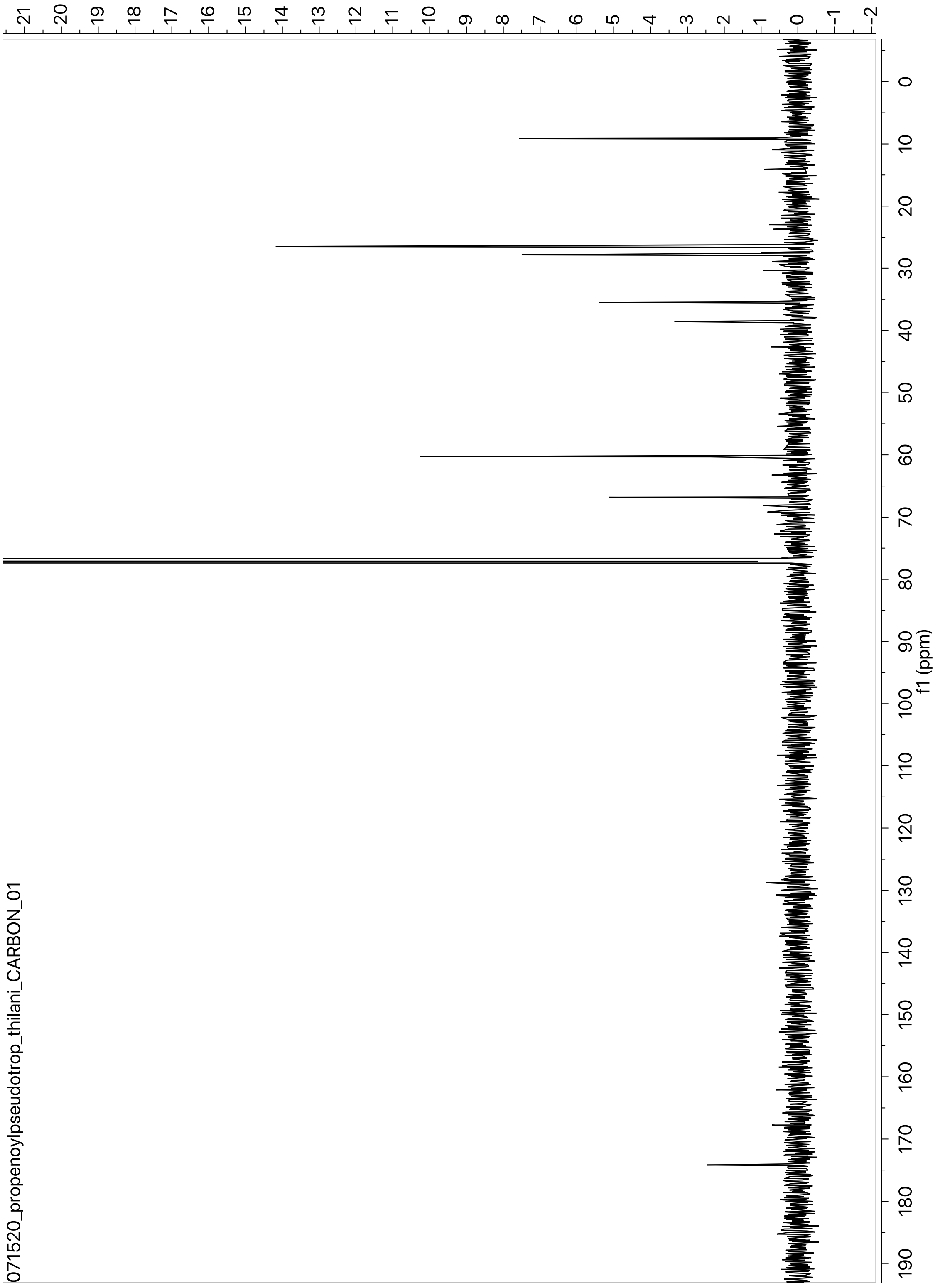

^{13}C NMR spectrum for 3-propionyl pseudotropine

C. 3-Isobutyryl pseudotropine

NMR chemical shifts values for 3-isobutyryl pseudotropine

<table>
<tr><td>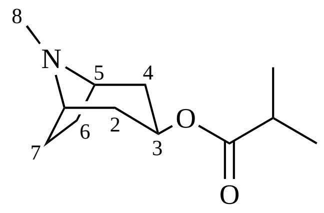</td><td colspan="2">8-methyl-8-azabicyclo[3.2.1]octan-3β-yl isobutyrate
(3-Isobutyryl pseudotropine)

Chemical Formula: $C_{12}H_{21}NO_2$
Experimental m/z of $[M+H]^+$: 212.1650
Theoretical m/z of $[M+H]^+$: 212.16451
InChI Key: UAINLAXRDPKCOO-JGPRNRPPSA-N
SMILES:
[H][C@]1(OC(C(C)C)=O)C[C@@H]2CC[C@@H](N2C)C
1
NMR (500 MHz, $CDCl_3$) ~2 mg</td></tr>
<tr><th>Carbon #
(group)</th><th>^{1}H (ppm)</th><th>^{13}C (ppm)</th></tr>
<tr><td>1,5 (CH)</td><td>3.22 (p, J = 5.2 Hz, 2H)</td><td>60.31</td></tr>
<tr><td>2,4 (CH_2)</td><td>Axial 1.74 (m, J = 15.2, 5.2 Hz, 2H)
Equatorial 1.83 (m, J = 15.2, 5.2 Hz, 2H)</td><td>35.50</td></tr>
<tr><td>3 (CH)
- 1 (CO)
- 2 (CH)
- 3,4 (CH_3)</td><td>4.97 (tt, J = 10.8, 6.4 Hz, 1H)
-
2.48 (hept, J = 7.0 Hz, 1H)
1.12 (d, J = 7.0 Hz, 6H)</td><td>66.69
176.13
34.07
18.94</td></tr>
<tr><td>6,7 (CH_2)</td><td>1.66 (m, J = 5.2, 9.5 Hz, 2H), 2.05 (m, J = 9.5 Hz, 2H)</td><td>26.46</td></tr>
<tr><td>8 N(CH_3)</td><td>2.33 (s, 3H)</td><td>40.04</td></tr>
</table>

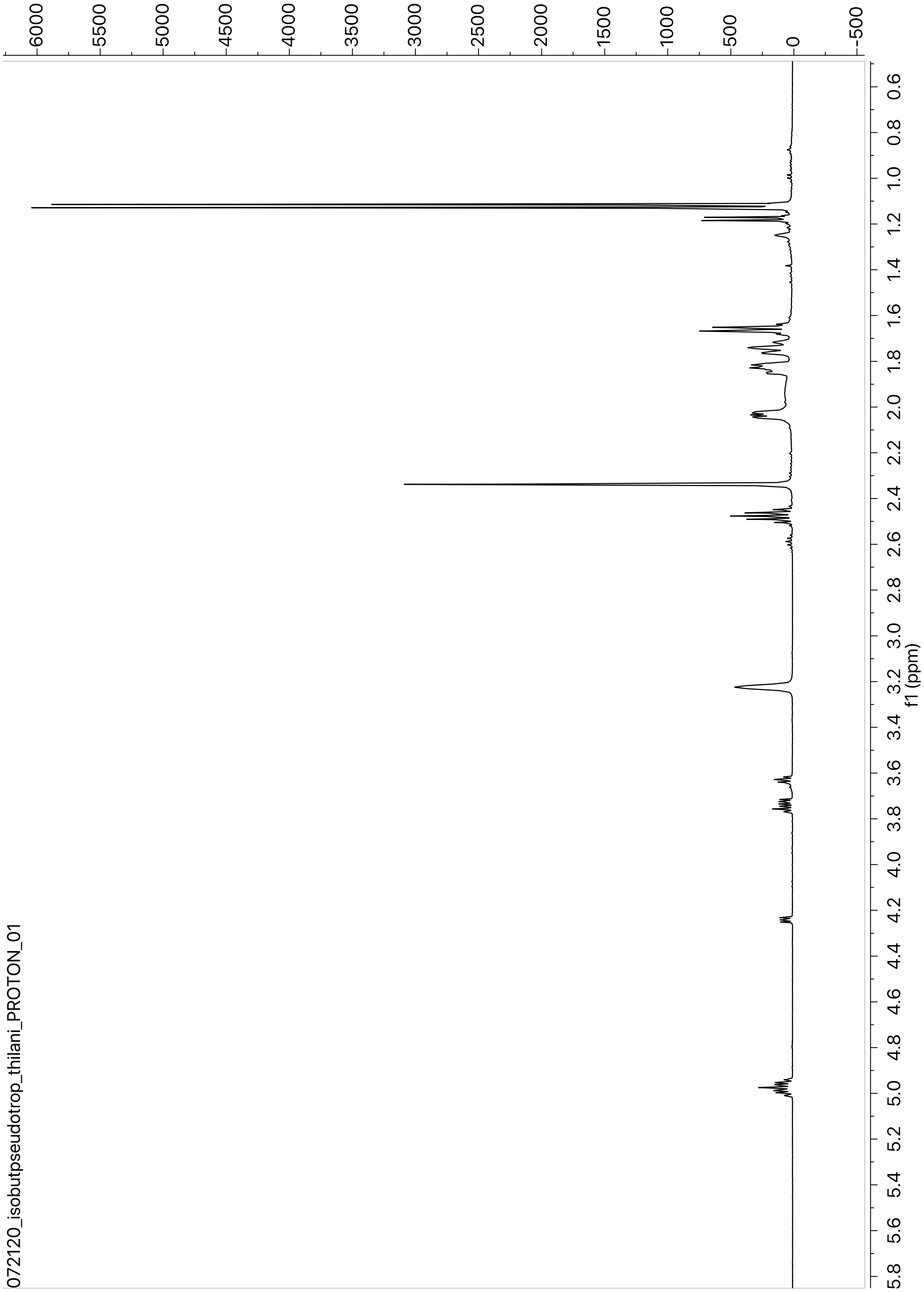

^{1}H NMR spectrum for 3-isobutyryl pseudotropine

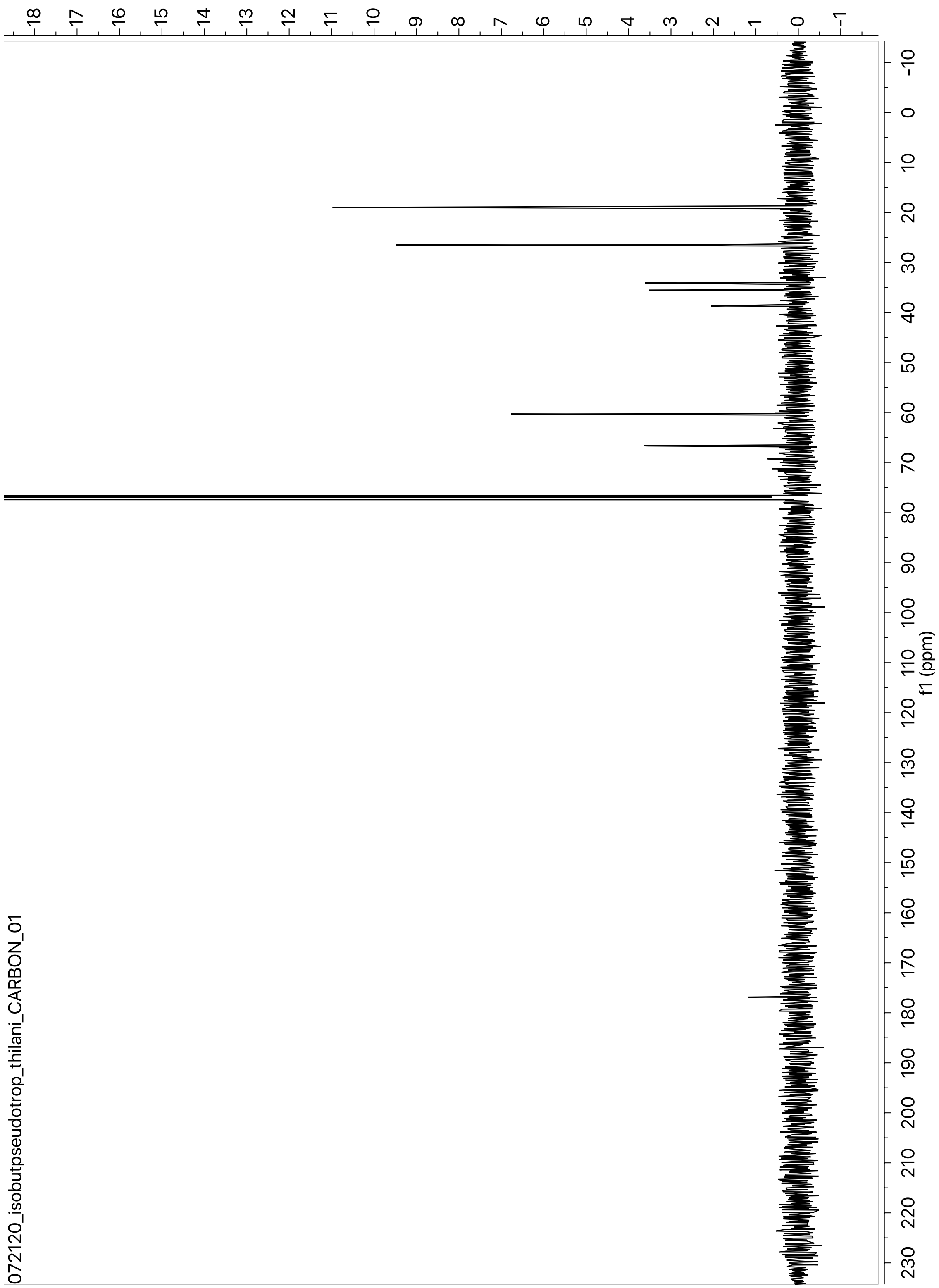

^{13}C NMR spectrum for 3-isobutyryl pseudotropine

D. <u>3-Isovaleryl pseudotropine</u>

NMR chemical shifts values for 3-isovaleryl pseudotropine

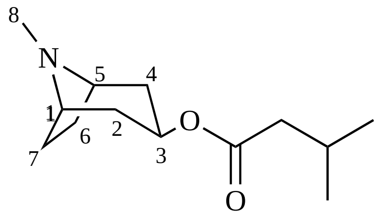

8-methyl-8-azabicyclo[3.2.1]octan-3β-yl 3-methylbutanoate
(3-Isovaleryl pseudotropine)

Chemical Formula: $C_{13}H_{23}NO_2$
Experimental *m/z*: 226.1807
Theoretical *m/z* of $[M+H]^+$: 226.18016
InChI Key: LXKKOLHRNROBTR-ZSBIGDGJSA-N
SMILES:
[H][C@]1(OC(CC(C)C)=O)C[C@@H]2CC[C@@H](N2C)C1
NMR (500 MHz, D_2O) ~10 mg

**Carbon # (group)**	**^{1}H (ppm)**	**^{13}C (ppm)**
**1,5** (CH)	3.82 (p, *J* = 6.2 Hz, 2H)	63.22
**2,4** (CH_2)	Axial 1.81 (m, *J* = 14.6, 10.9 Hz, 2H)<br>Equatorial 2.15 (m, *J* = 14.6, 6.2 Hz, 2H)	34.65
**3** (CH)<br>- 1 (CO)<br>- 2 (CH_2)<br>- 3 (CH)<br>- 4,5 (CH_3)	5.02 (tt, *J* = 10.9, 6.2 Hz, 1H)<br>-<br>2.09 (d, *J* = 7.0 Hz, 2H)<br>1.88 (m, *J* = 7.0 Hz, 1H)<br>0.75 (d, *J* = 7.0 Hz, 6H)	64.25<br>175.60<br>34.91<br>25.33<br>21.38
**6,7** (CH_2)	1.95 (m, *J* = 6.2 Hz, 2H), 2.20 (m, *J* = 6.2 Hz, 2H)	23.56
**8** N(CH_3)	2.61 (s, 3H)	37.98

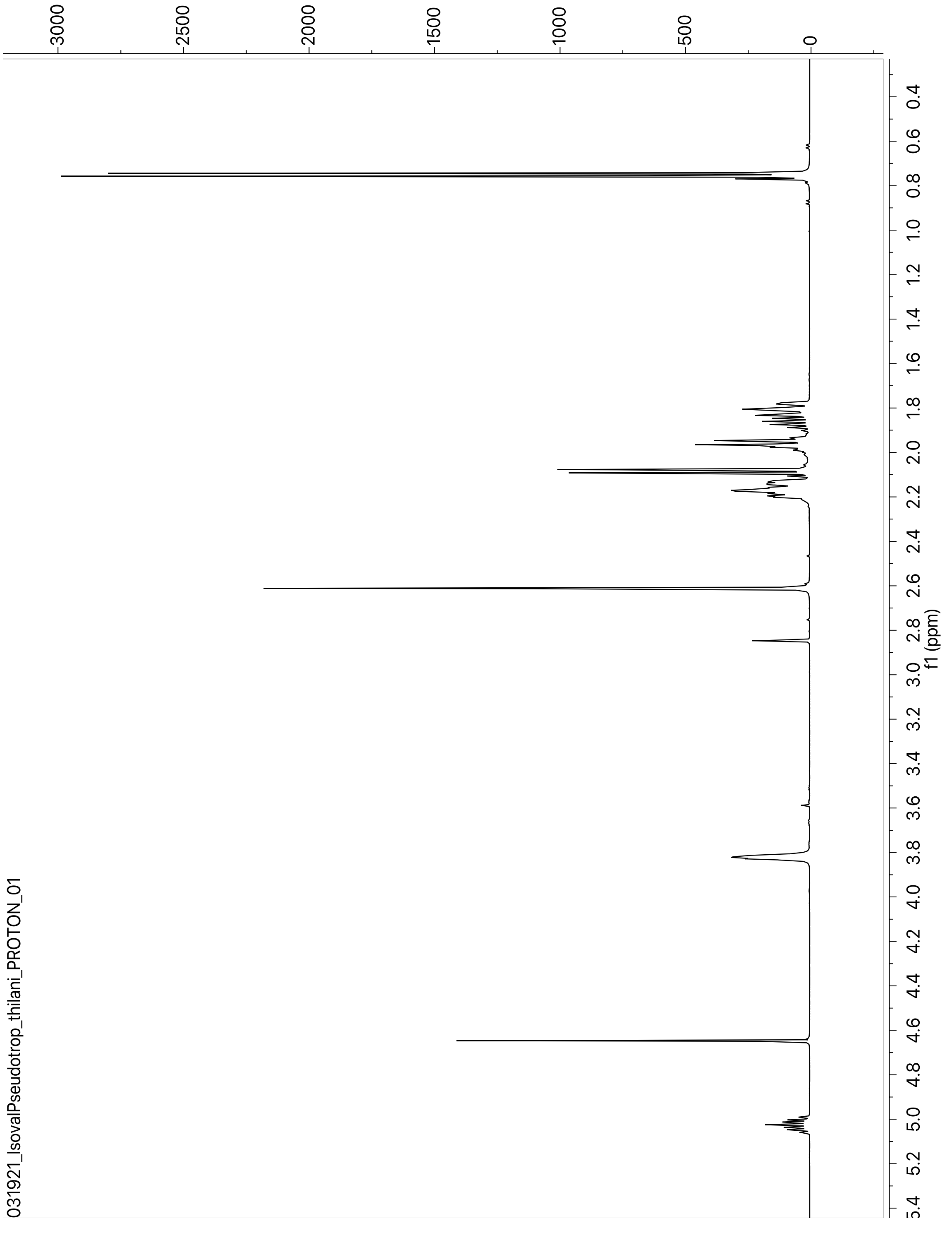

¹H NMR spectrum for 3-isovaleryl pseudotropine

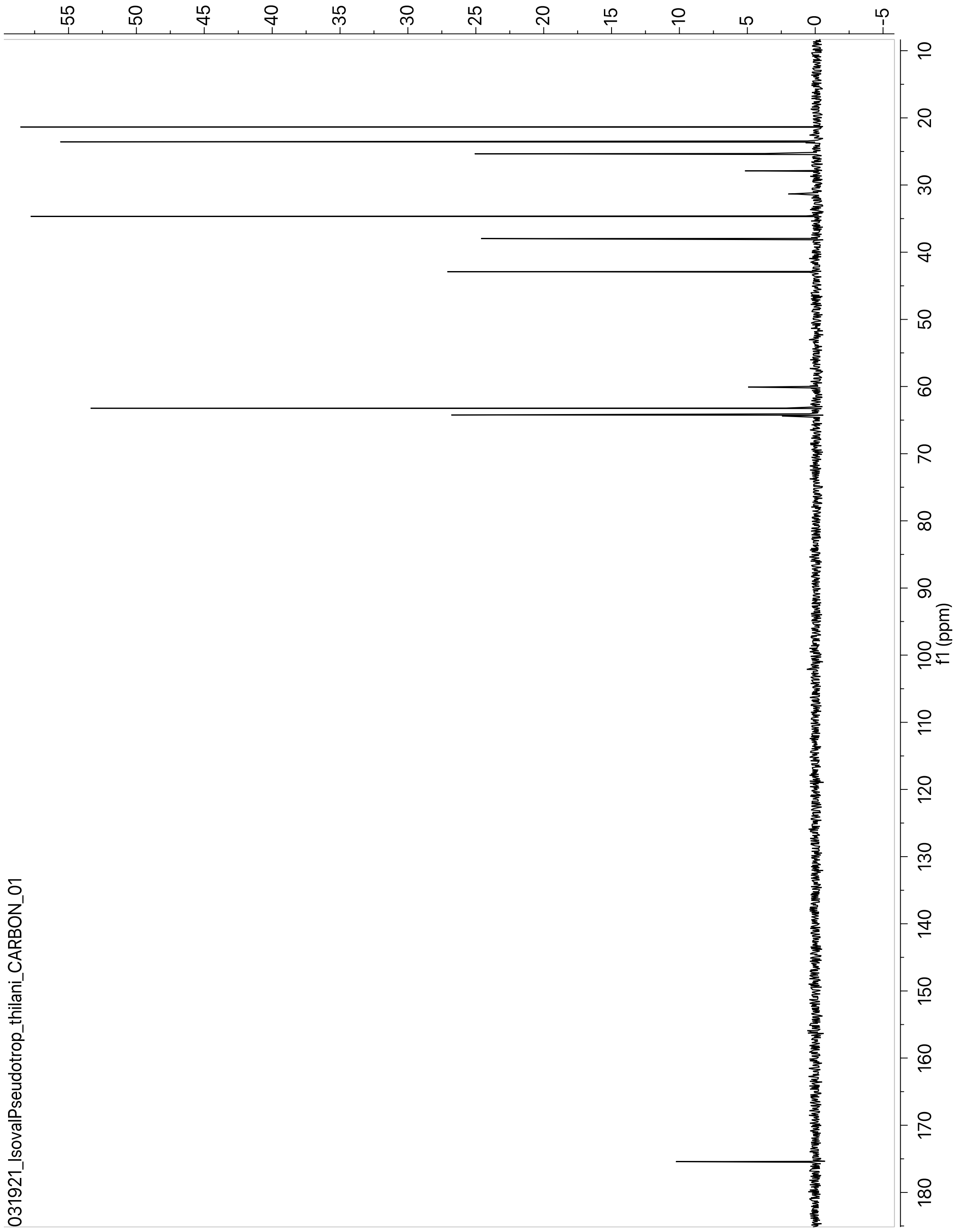

^{13}C NMR spectrum for 3-isovaleryl pseudotropine

E. 3-Senecioyl pseudotropine

NMR chemical shift values for 3-senecioyl pseudotropine

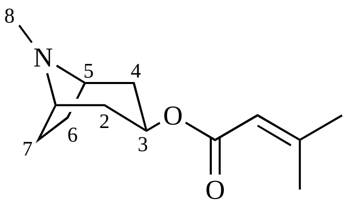

8-methyl-8-azabicyclo[3.2.1]octan-3β-yl 3-methylbut-2-enoate
(3-Senecioyl pseudotropine)

Chemical Formula: $C_{13}H_{21}NO_2$
Experimental *m/z* of $[M+H]^+$: 224.1645
Theoretical *m/z* of $[M+H]^+$: 224.16451
InChI Key: PFQFRMFXPJMNJJ-ZSBIGDGJSA-N
SMILES:
[H][C@]1(OC(/C=C(C)\C)=O)C[C@@H]2CC[C@@H](N2C)C1
NMR (500 MHz, $CDCl_3$) ~2 mg

**Carbon # (group)**	**^{1}H (ppm)** [a]from HSQC	**^{13}C (ppm)** [b]from HMBC
**1,5** (CH)	3.26 (m, *J* = 5.2 Hz, 2H)	60.39
**2,4** (CH_2)	Axial 1.83[a] (m, *J* = 5.5, 11.4 Hz, 2H)<br>Equatorial 1.88[a] (m, *J* = 5.2, 11.4 Hz, 2H)	35.40
**3** (CH)<br>- 1 (CO)<br>- 2 (CH)<br>- 3(C)<br>- 4(CH_3)<br>- 5(CH_3)	5.04 (t, *J* = 5.4 Hz, 1H)<br>-<br>5.64 (hept, *J* = 1.3 Hz, 1H)<br>-<br>2.17 (m, *J* = 2.6, 1.3 Hz, 3H)<br>1.90 (m, *J* = 12.6, 1.3 Hz, 3H)	65.71<br>166.47<br>116.30<br>156.70[b]<br>20.16<br>27.43
**6,7** (CH_2)	1.68 (m, *J* =5.2 Hz, 2H), 2.06 (m, *J* = 11.9, 5.2 Hz, 2H)	26.34
**8** N(CH_3)	2.37 (s, 3H)	38.44

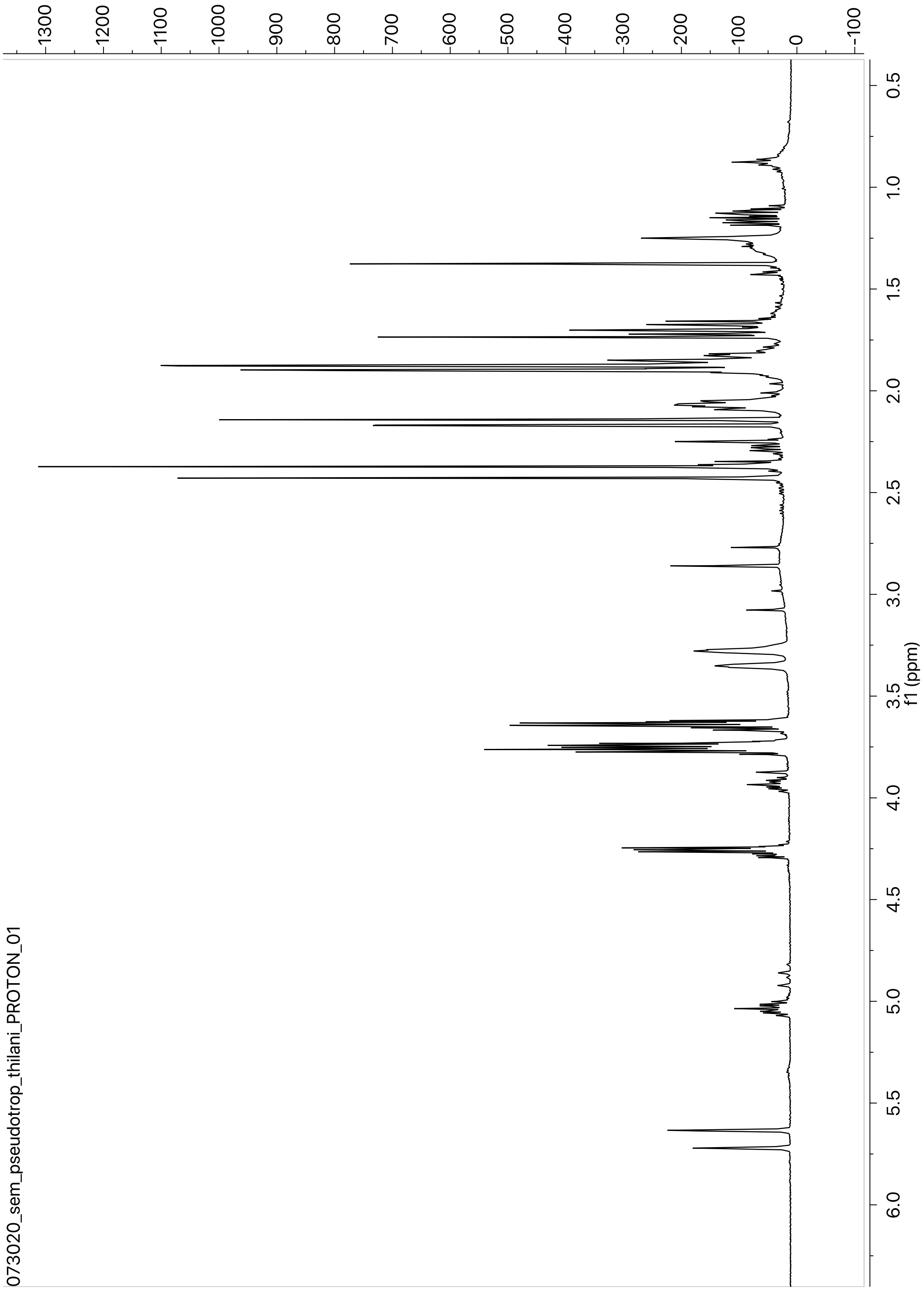

¹H NMR spectrum for 3-senecioyl pseudotropine

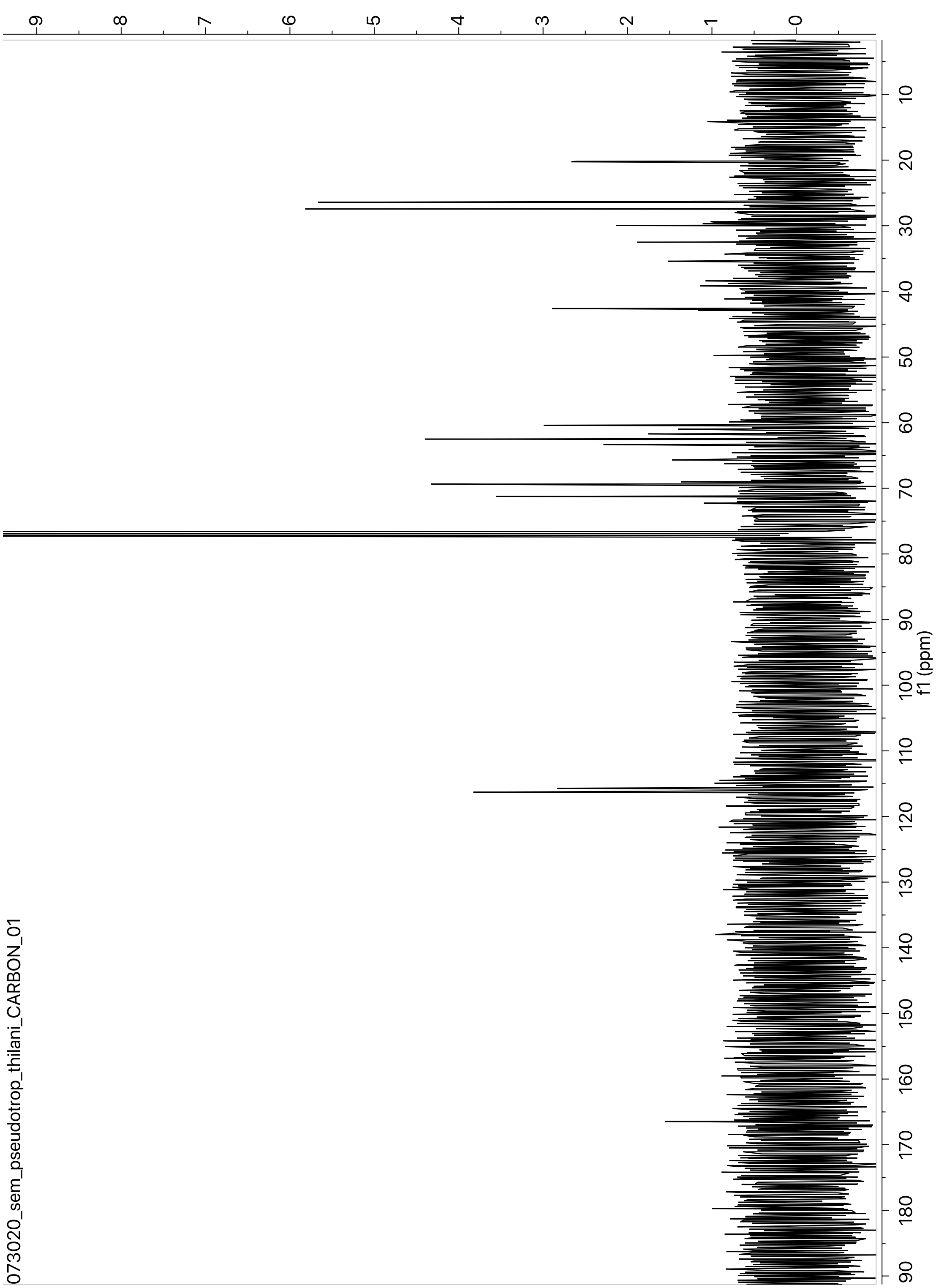

^{13}C NMR spectrum for 3-senecioyl pseudotropine

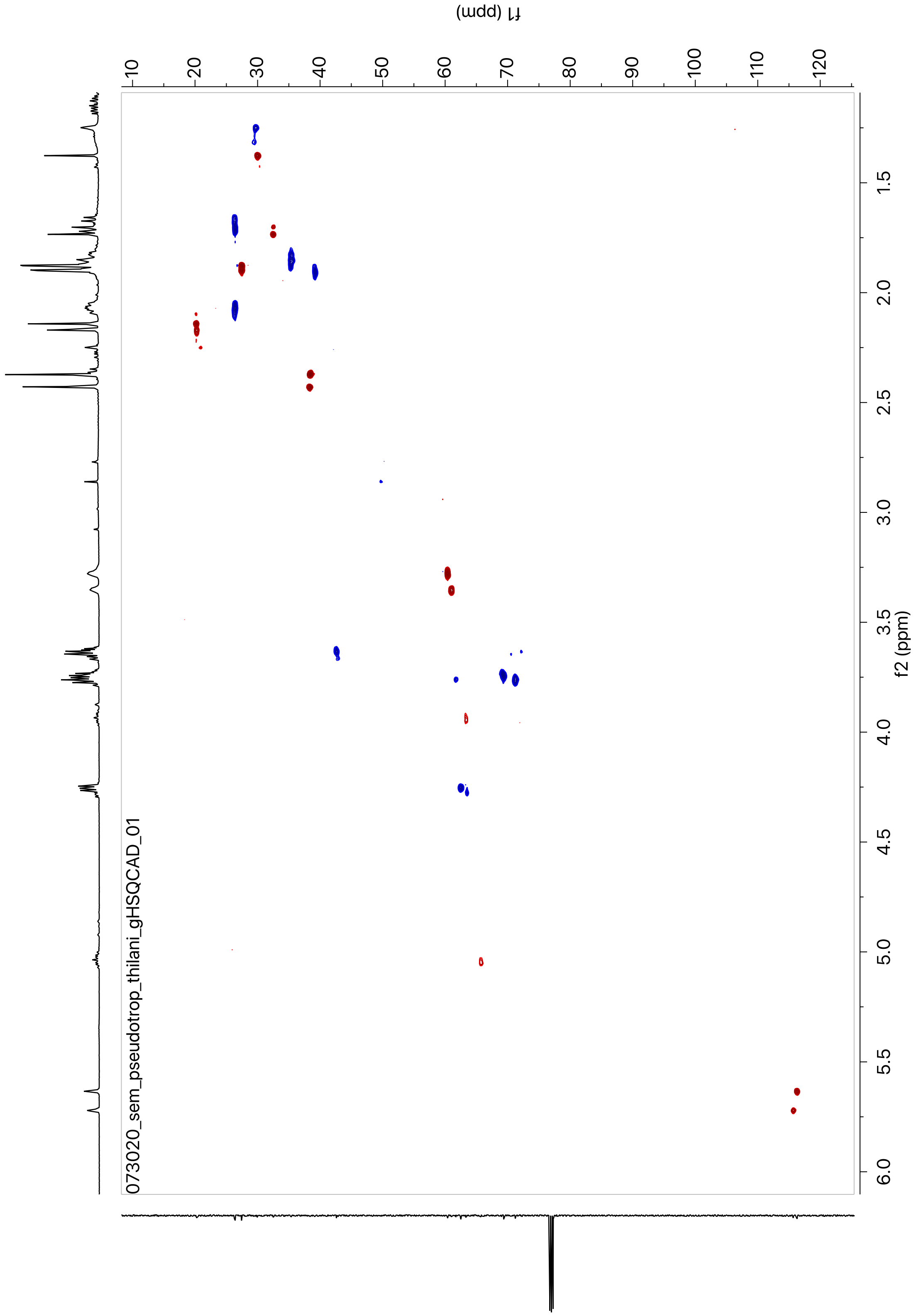

gHSQCAD spectrum for 3-senecioyl pseudotropine

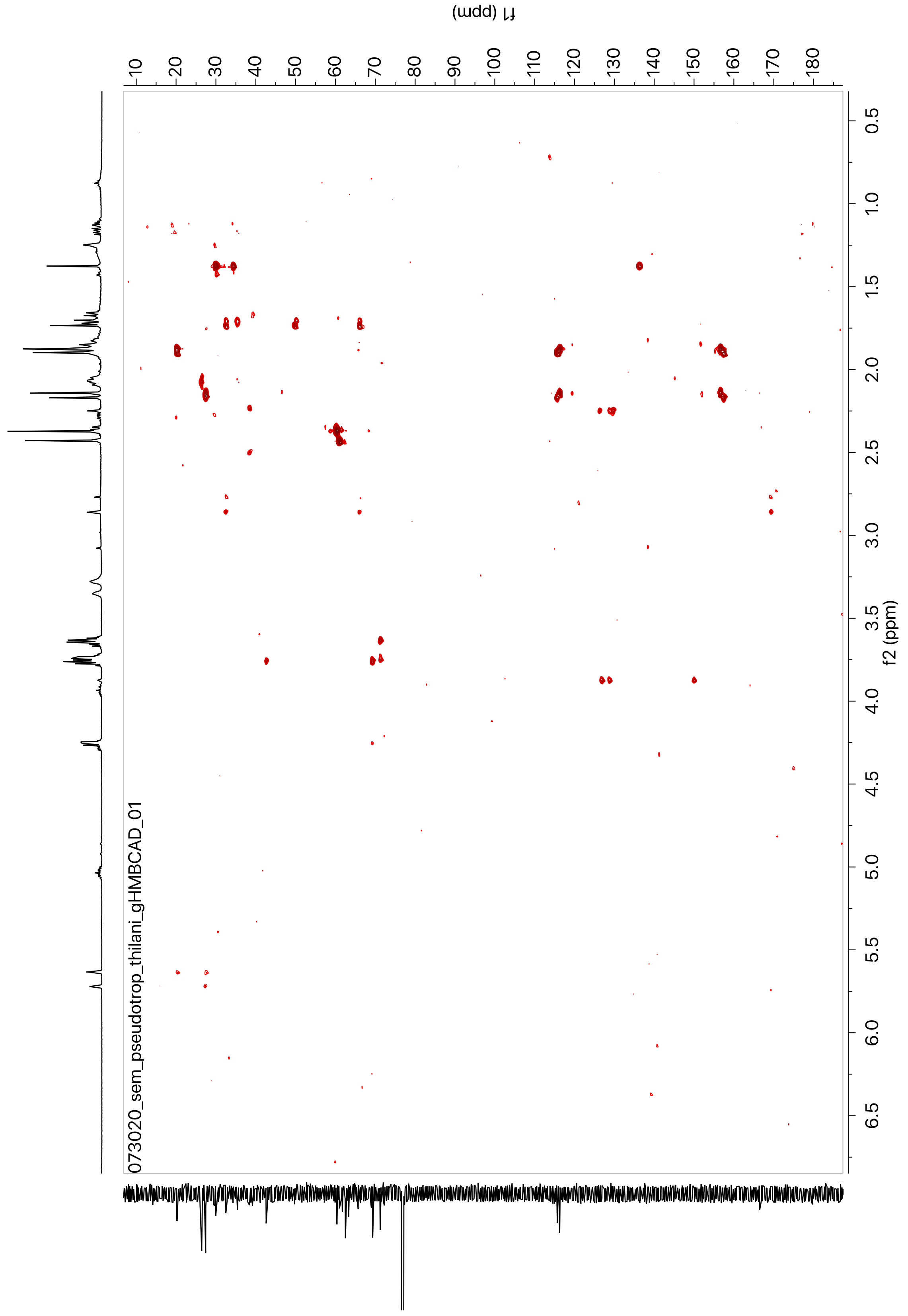

gHMBCAD spectrum for 3-senecioyl pseudotropine

F. **3-Tigloyl pseudotropine**

NMR chemical shift values for 3-tigloyl pseudotropine

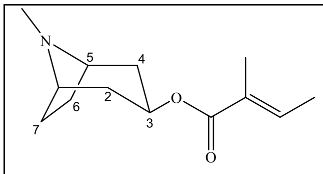

8-methyl-8-azabicyclo[3.2.1]octan-3β-yl (*E*)-2-methylbut-2-enoate
(3-Tigloyl pseudotropine)

Chemical Formula: $C_{13}H_{21}NO_2$
Experimental *m/z* of $[M+H]^+$: 224.1659
Theoretical *m/z* of $[M+H]^+$: 224.16451
InChI Key: UVHGSMZRSVGWDJ-WJKWMCMVSA-N
SMILES:
[H][C@]1(OC(/C(C)=C/C)=O)C[C@@H]2CC[C@@H](N2C)C1
NMR (500 MHz, D_2O) ~2 mg

**Carbon # (group)**	**^{1}H (ppm)**	**^{13}C (ppm)**
**1,5** (CH)	3.97 (m, 2H)	63.27
**2,4** (CH_2)	Axial 1.98 (ddd, J = 14.1, 10.9, 2.8 Hz, 2H)<br>Equatorial 2.32 (m, 2H)	34.73
**3** (CH)<br>- 1 (CO)<br>- 2 (C)<br>- 3 (CH_3)<br>- 4 (CH)<br>- 5 (CH_3)	5.18 (tt, J = 11.2, 6.2 Hz, 1H)<br>-<br>-<br>1.77 (d, J = 6.3 Hz, 3H)<br>6.90 (m, 1H)<br>1.77 (d, J = 6.3 Hz, 3H)	64.29<br>169.47<br>127.52<br>11.00<br>140.26<br>13.69
**6,7** (CH_2)	2.32 (tq, J = 14.9, 5.9, 4.5 Hz, 2H), 2.13 (m, 2H)	23.57
**8** N(CH_3)	2.76 (s, 3H)	37.96

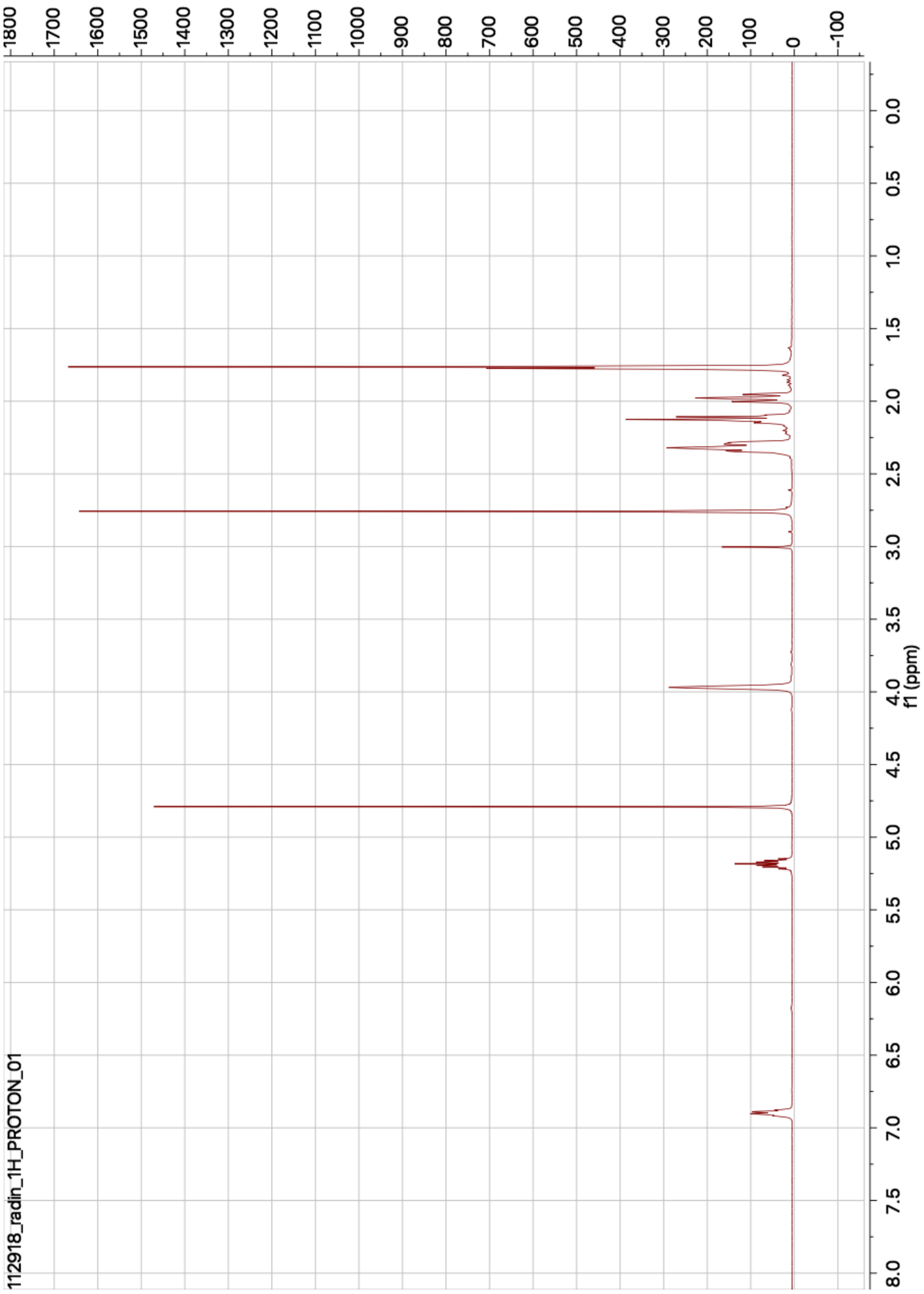

¹H NMR spectrum for 3-tigloyl pseudotropine

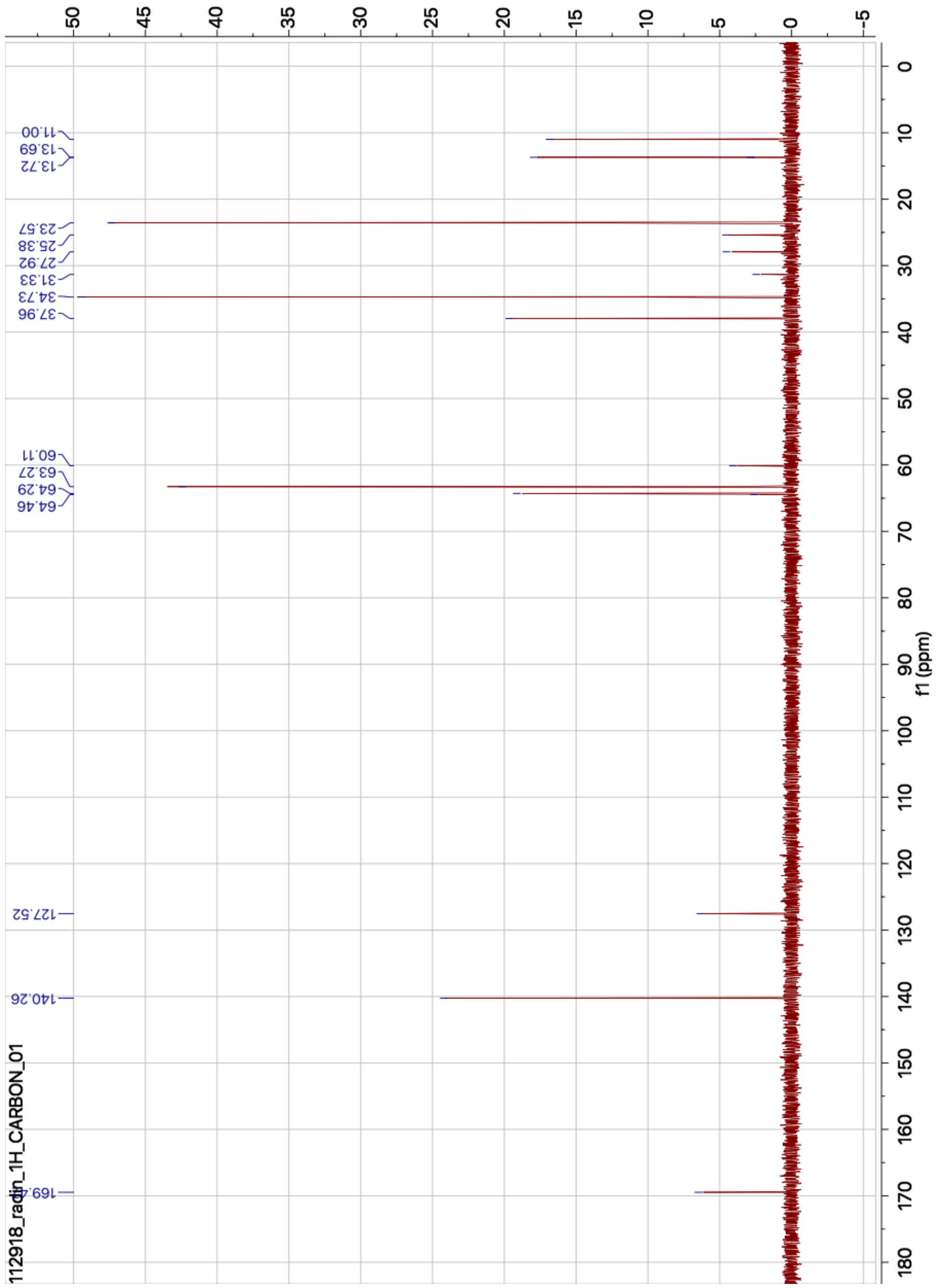

^{13}C NMR spectrum for 3-tigloyl pseudotropine

O-ACYL TROPINE

G. 3-Acetyl tropine

NMR chemical shifts values for 3-acetyl tropine

<table>
<tr><td>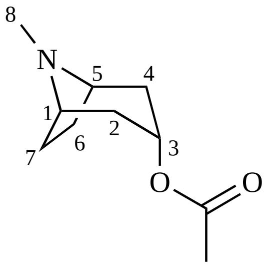
</td><td colspan="2">8-methyl-8-azabicyclo[3.2.1]octan-3α-yl acetate
(3-Acetyl tropine)

Chemical Formula: $C_{10}H_{17}NO_2$
Experimental m/z of $[M+H]^+$: 184.1333
Theoretical m/z of $[M+H]^+$:184.13321
InChI Key: MDIDMOWWLBGYPG-MYJAWHEDSA-N
SMILES:
[H][C@@]1(OC(C)=O)C[C@@H]2CC[C@@H](N2C)C1
NMR (500 MHz, D_2O) ~2 mg</td></tr>
<tr><th>Carbon #
(group)</th><th>^{1}H (ppm)</th><th>^{13}C (ppm)</th></tr>
<tr><td>1,5 (CH)</td><td>3.74 (m, J = 5.0 Hz, 2H)</td><td>62.24</td></tr>
<tr><td>2,4 (CH_2)</td><td>Axial 1.98 (dd, J = 16.5, 4.9 Hz, 2H)
Equatorial 3.08 (dt, J = 16.5, 4.9 Hz, 2H)</td><td>34.57</td></tr>
<tr><td>3 (CH)
- 1 (CO)
- 2 (CH_3)</td><td>5.14 (t, J = 4.9 Hz, 1H)
-
2.07 (s, 3H)</td><td>64.65
169.47
21.36</td></tr>
<tr><td>6,7 (CH_2)</td><td>2.24 (dd, J = 7.4, 5.0 Hz, 2H), 2.39 (d, J = 7.4 Hz, 2H)</td><td>24.44</td></tr>
<tr><td>8 N(CH_3)</td><td>2.75 (d, J = 5.0 Hz, 3H)</td><td>39.26</td></tr>
</table>

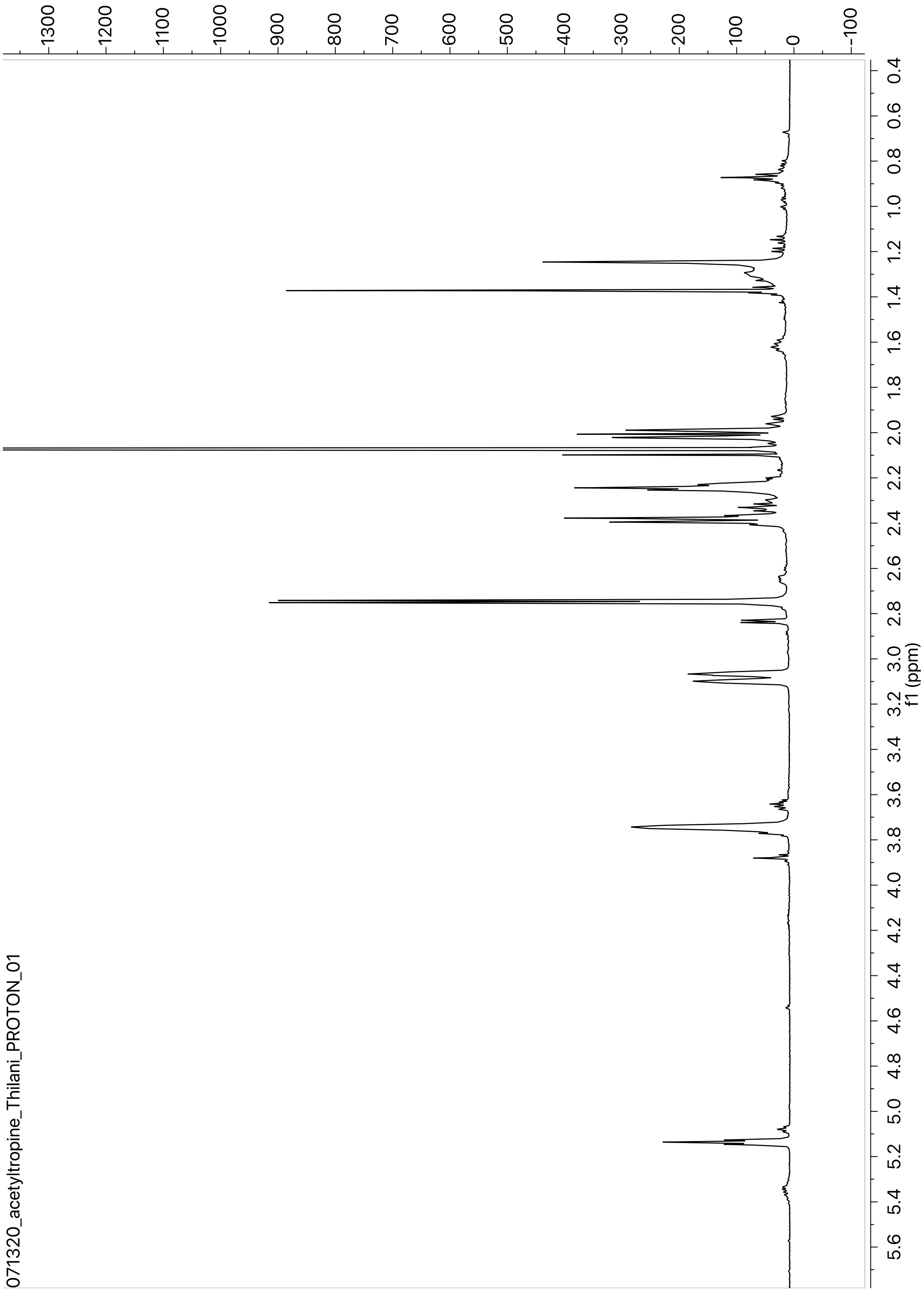

^{1}H NMR spectrum for 3-acetyl tropine

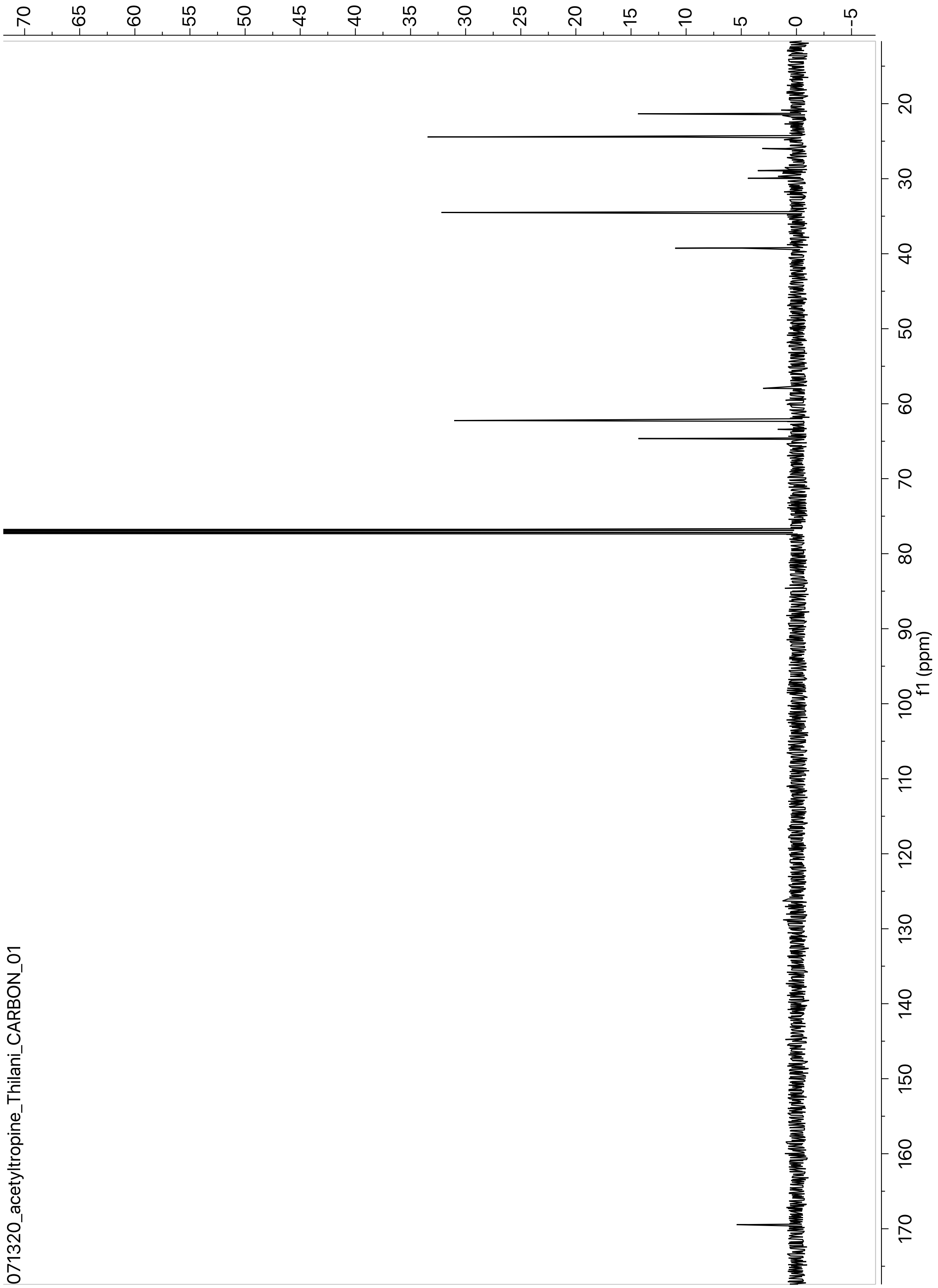

^{13}C NMR spectrum for 3-acetyl tropine

H. 3-Propionyl tropine

NMR chemical shifts values for 3-propionyl tropine

<table>
<tr><td>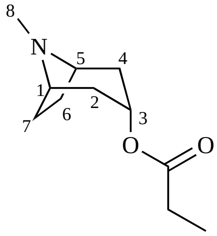
</td><td colspan="2">8-methyl-8-azabicyclo[3.2.1]octan-3α-yl propionate
(3-Propionyl tropine)

Chemical Formula: $C_{11}H_{19}NO_2$
Experimental *m/z* of $[M+H]^+$: 198.1493
Theoretical *m/z* of $[M+H]^+$: 198.14886
InChI Key: NUFSBXOAMBFLRJ-MYJAWHEDSA-N
SMILES:
[H][C@@]1(OC(CC)=O)C[C@@H]2CC[C@@H](N2C)C1
NMR (500 MHz, $CDCl_3$) ~2 mg</td></tr>
<tr><th>Carbon #
(group)</th><th>^{1}H (ppm)</th><th>^{13}C (ppm)
From HMBC
and HSQC</th></tr>
<tr><td>**1,5** (CH)</td><td>3.30 (m, *J* = 5.3 Hz, 2H)</td><td>60.11</td></tr>
<tr><td>**2,4** (CH_2)</td><td>Axial 1.76 (d, *J* = 15.2 Hz, 2H)
Equatorial 1.83 (dt, *J* = 15.2, 5.6 Hz, 2H)</td><td>35.71</td></tr>
<tr><td>**3** (CH)
- 1 (CO)
- 2 (CH_2)
- 2 (CH_3)</td><td>5.03 (t, *J* = 5.3 Hz, 1H)
-
2.32 (q, *J* = 7.6 Hz, 2H)
1.16 (t, *J* = 7.6 Hz, 3H)</td><td>66.38
173.83
28.20
9.23</td></tr>
<tr><td>**6,7** (CH_2)</td><td>1.64 (m, *J* = 5.3 Hz, 2H), 2.07 (m, *J* = 5.3 Hz, 2H)</td><td>26.50</td></tr>
<tr><td>**8** N(CH_3)</td><td>2.41 (s, 3H)</td><td>39.57</td></tr>
</table>

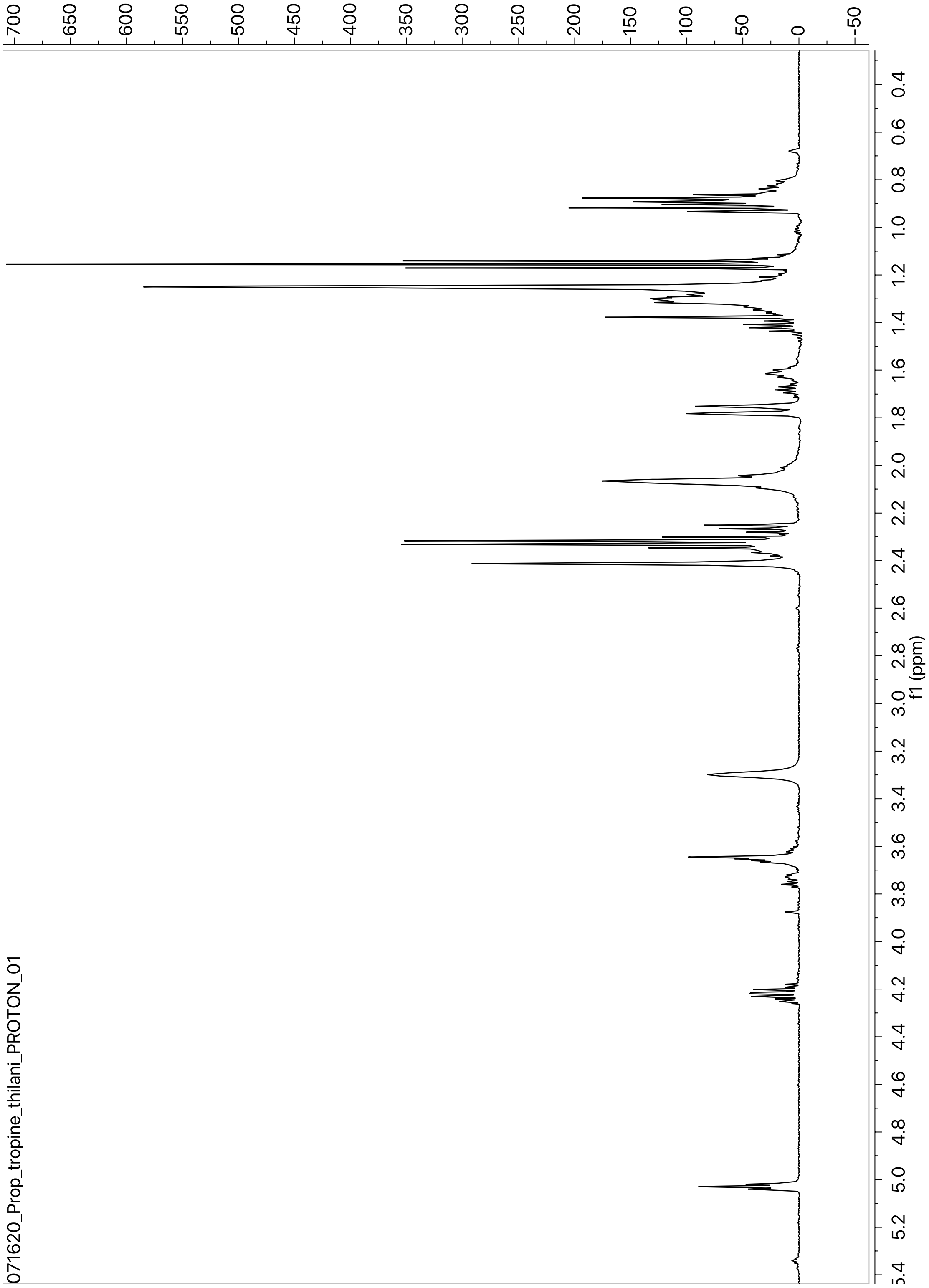

^{1}H NMR spectrum for 3-propionyl tropine

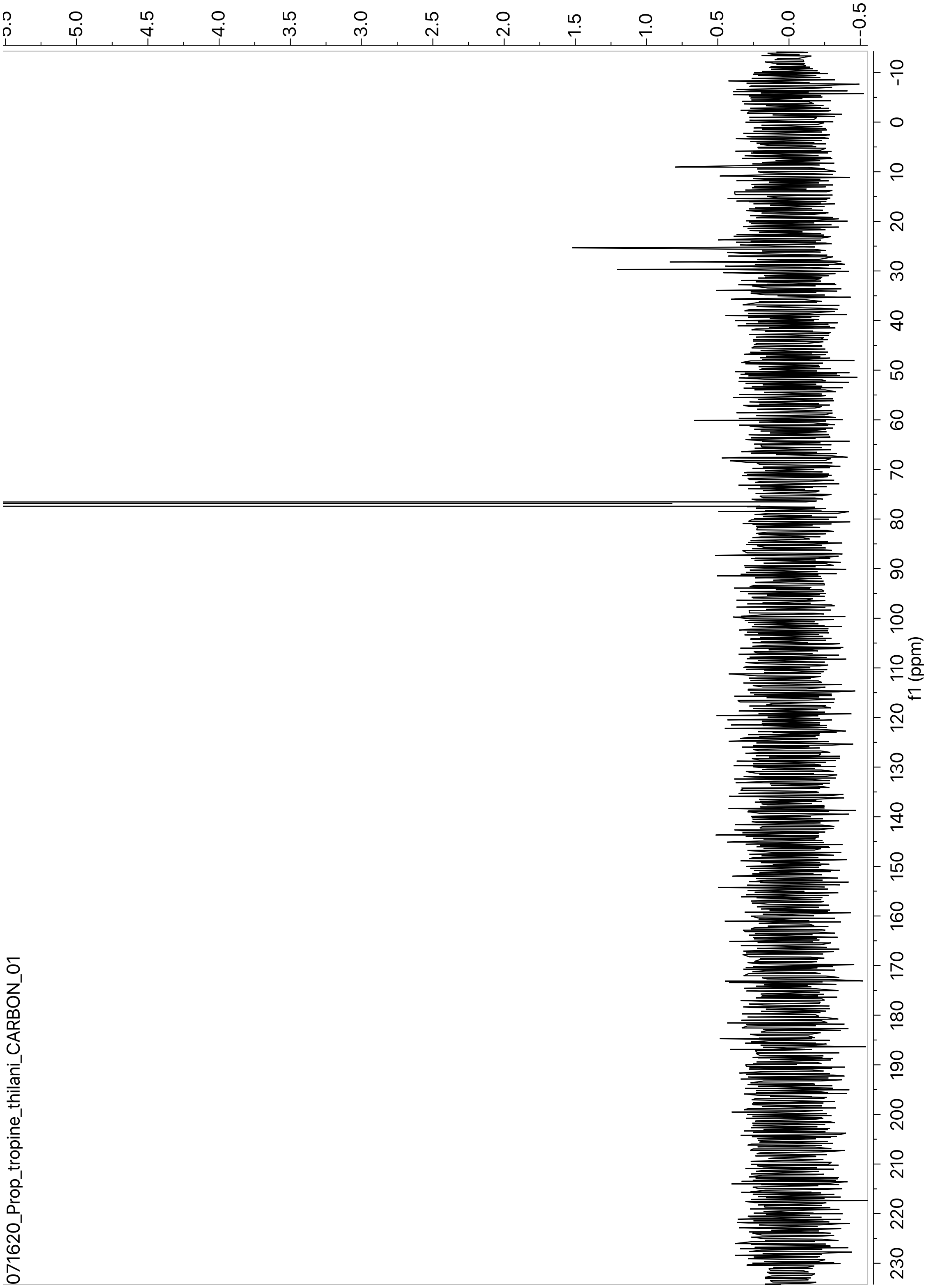

^{13}C NMR spectrum for 3-propionyl tropine

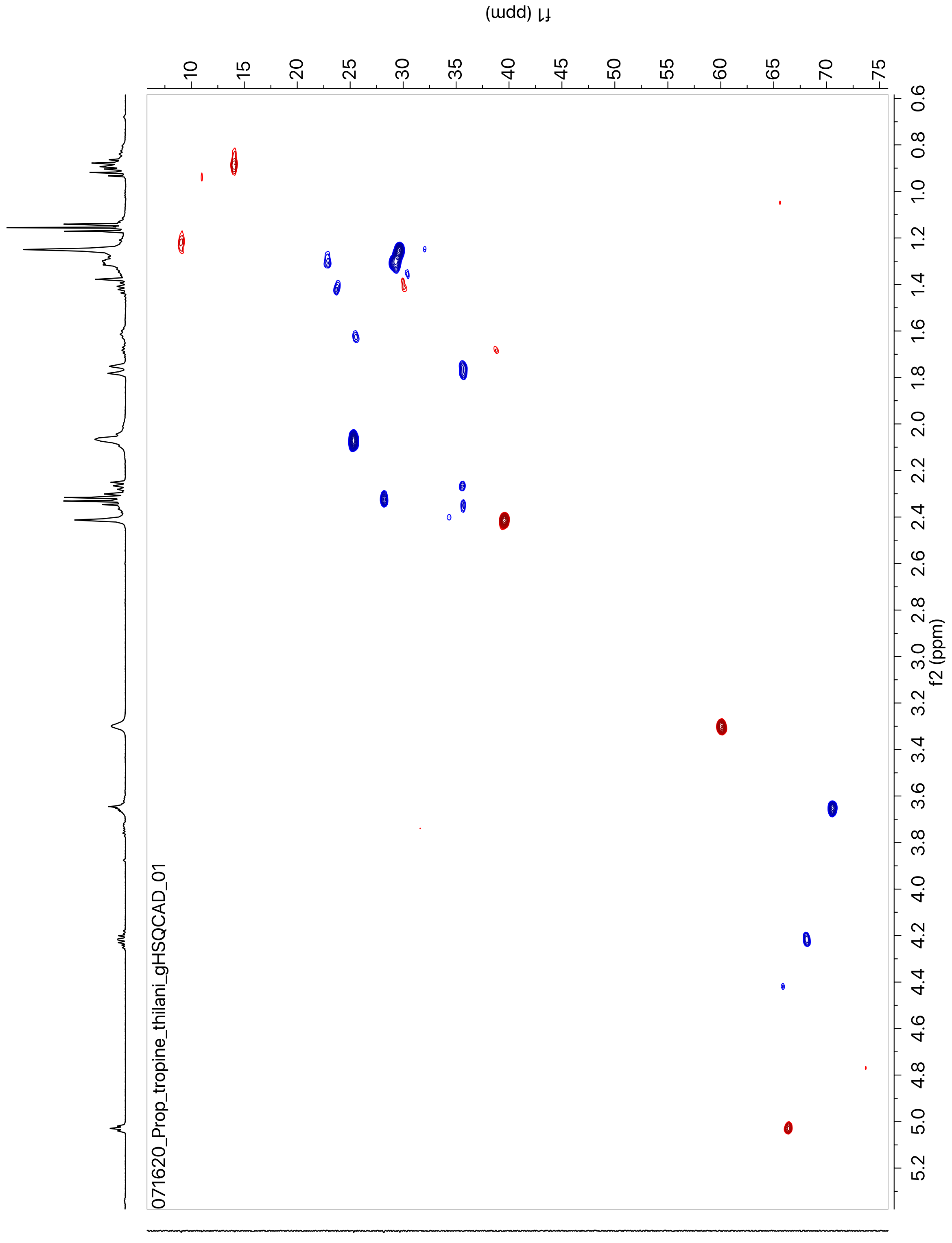

gHSQCAD NMR spectrum for 3-propionyl tropine

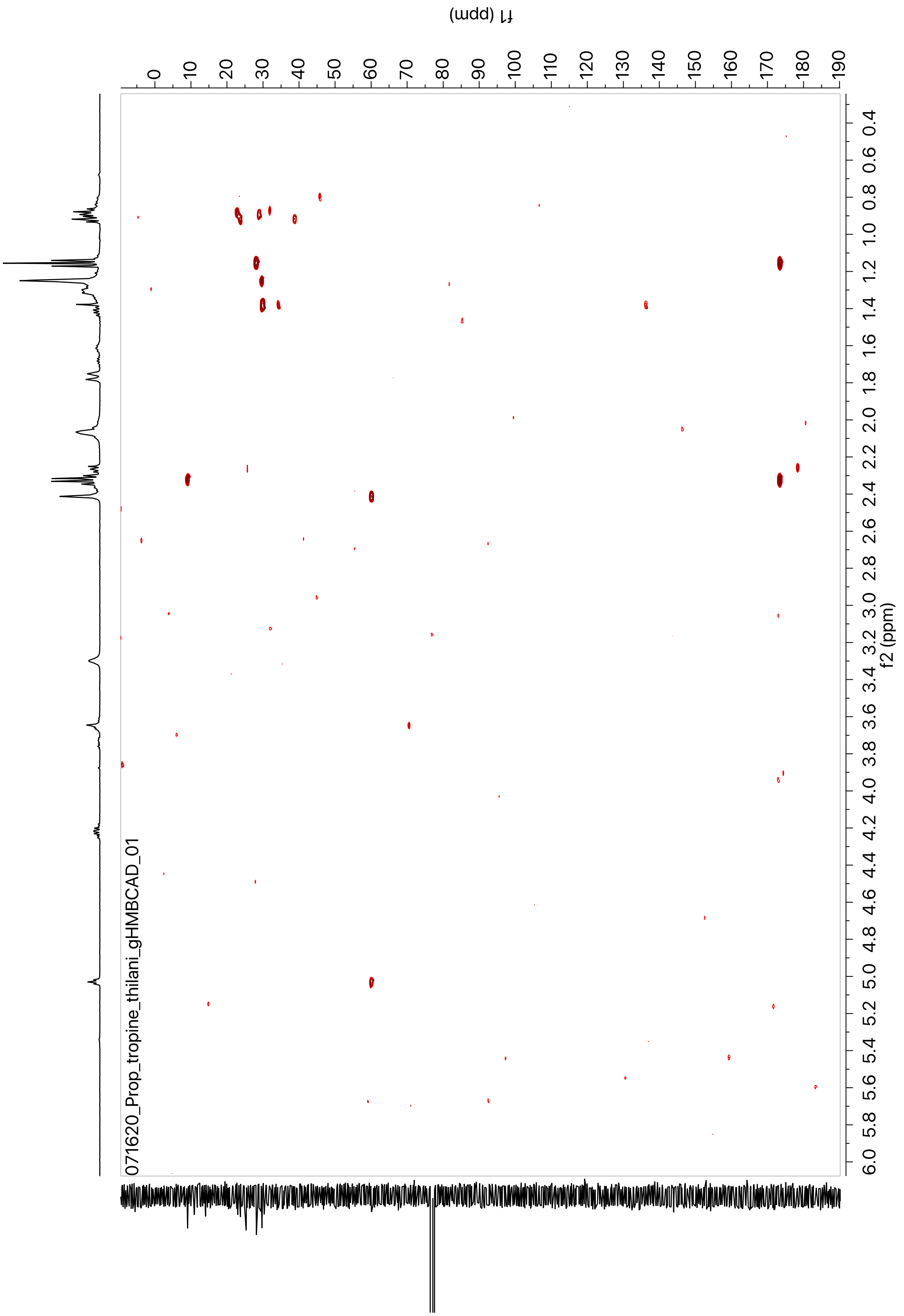

gHMBCAD NMR spectrum for 3-propionyl tropine

I. 3-Isobutyryl tropine

NMR chemical shift values for 3-isobutyryl tropine

<table>
<tr>
<td>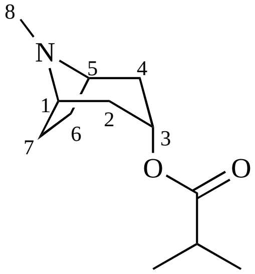</td>
<td colspan="2">8-methyl-8-azabicyclo[3.2.1]octan-3α-yl isobutyrate
(3-Isobutyryl tropine)

Chemical Formula: $C_{12}H_{21}NO_2$
Experimental m/z of $[M+H]^+$: 212.1648
Theoretical m/z of $[M+H]^+$: 212.16451
InChI Key: UAINLAXRDPKCOO-URLYPYJESA-N
SMILES:
[H][C@@]1(OC(C(C)C)=O)C[C@@H]2CC[C@@H](N2C)
C1
NMR (500 MHz, $CDCl_3$) ~2 mg</td>
</tr>
<tr>
<th>Carbon #
(group)</th>
<th>^{1}H (ppm)</th>
<th>^{13}C (ppm)</th>
</tr>
<tr>
<td>1,5 (CH)</td>
<td>3.18 (m, J = 5.2 Hz, 2H)</td>
<td>59.83</td>
</tr>
<tr>
<td>2,4 (CH_2)</td>
<td>Axial 1.71 (d, J = 15.2 Hz, 2H)
Equatorial 2.23 (m, J = 15.2, 5.6 Hz, 2H)</td>
<td>36.25</td>
</tr>
<tr>
<td>3 (CH)
- 1 (CO)
- 2 (CH)
- 3,4 (CH_3)</td>
<td>4.99 (t, J = 5.5 Hz, 1H)
-
2.51 (hept, J = 7.0 Hz, 1H)
1.18 (d, J = 7.0 Hz, 6H)</td>
<td>66.72
176.13
34.36
18.87</td>
</tr>
<tr>
<td>6,7 (CH_2)</td>
<td>1.99 (m, J = 5.2, 9.5 Hz, 2H), 2.04 (m, J = 5.2 Hz, 2H)</td>
<td>25.49</td>
</tr>
<tr>
<td>8 N(CH_3)</td>
<td>2.33 (s, 3H)</td>
<td>40.04</td>
</tr>
</table>

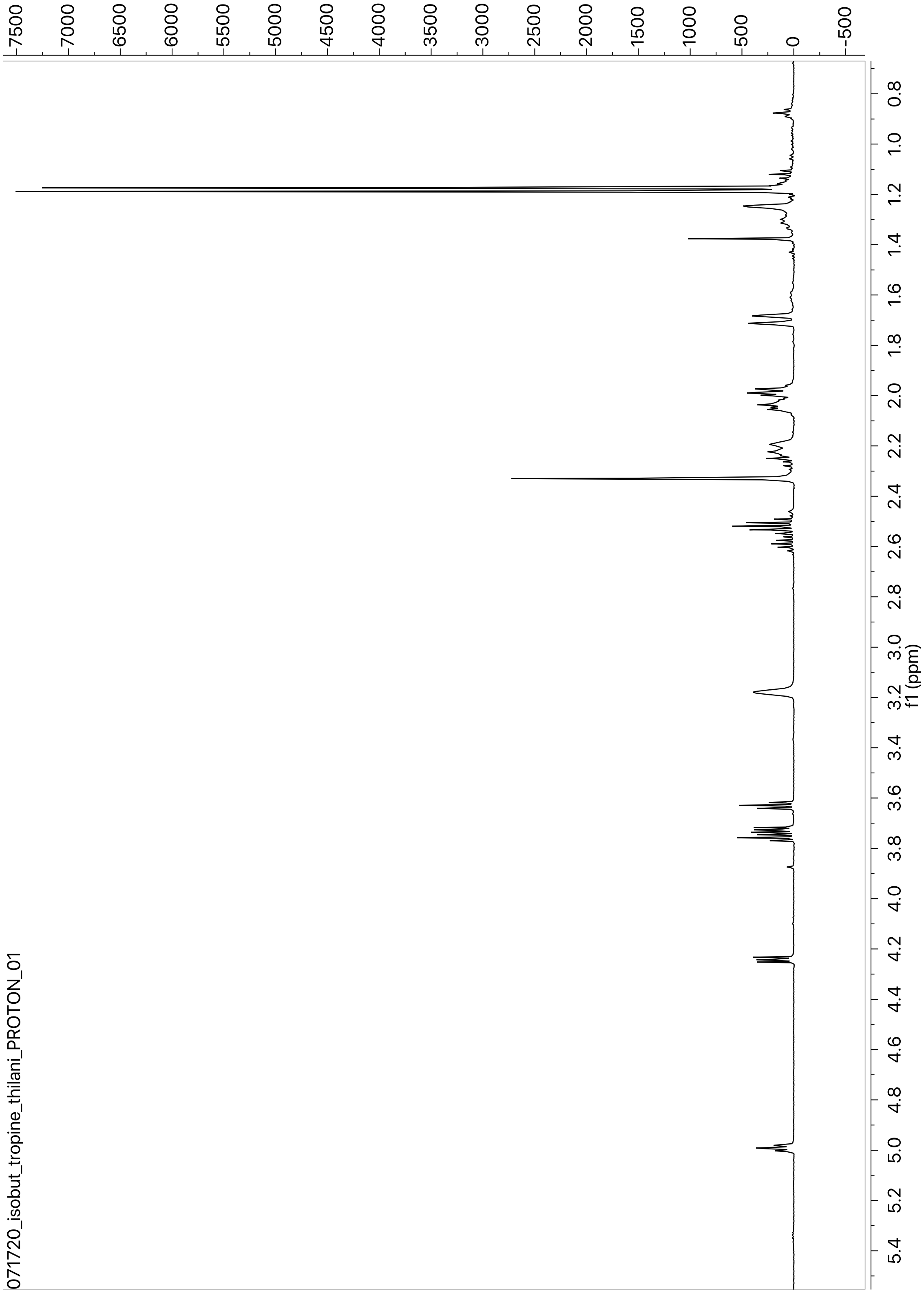

^{1}H NMR spectrum for 3-isobutyryl tropine

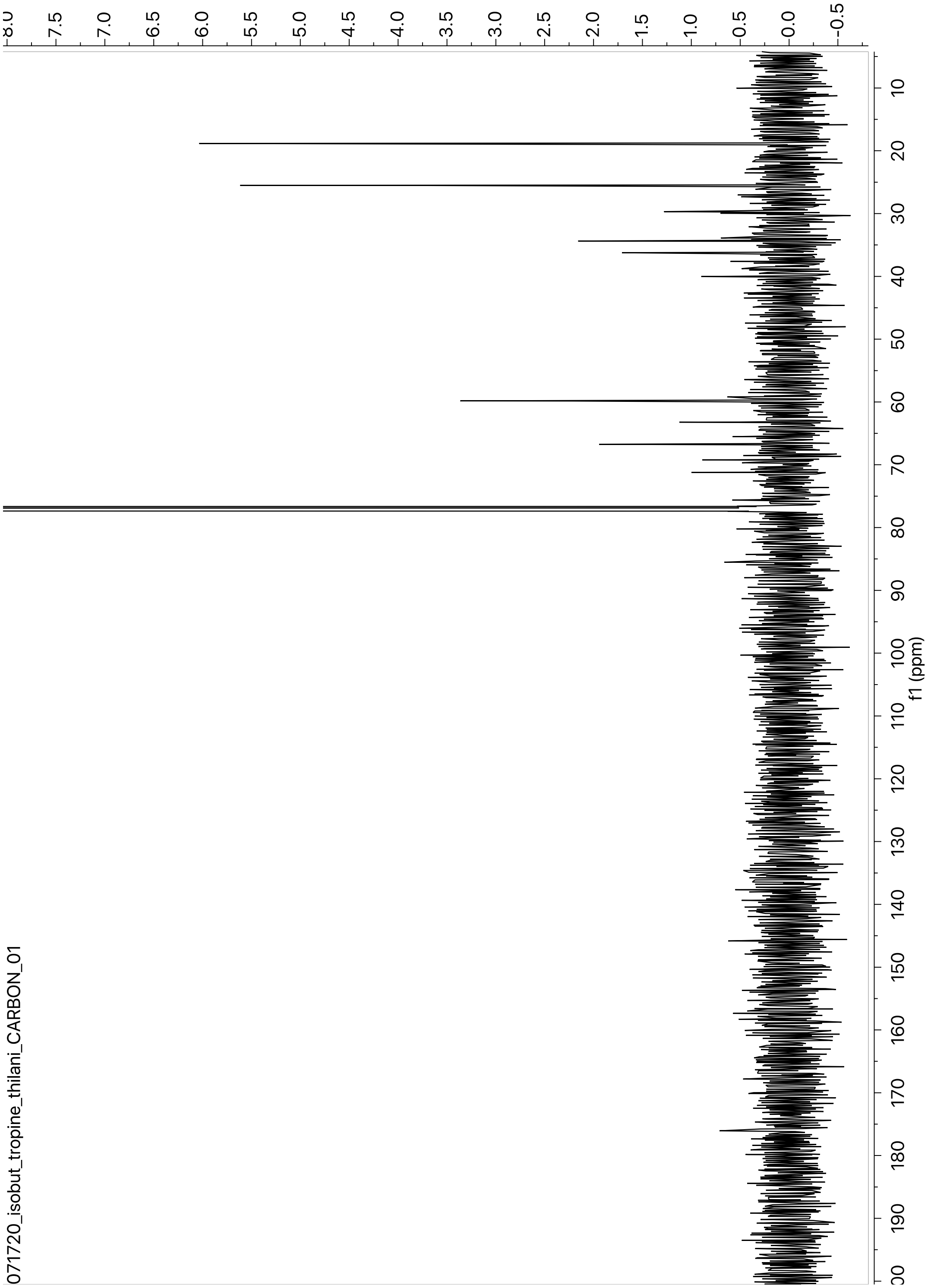

^{13}C NMR spectrum for 3-isobutyryl tropine

J. <u>3-Isovaleryl tropine</u>

NMR chemical shift values for 3-isovaleryl tropine

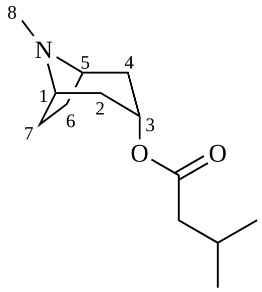	8-methyl-8-azabicyclo[3.2.1]octan-3α-yl 3-methylbutanoate<br>(3-Isovaleryl tropine)<br><br>Chemical Formula: $C_{13}H_{23}NO_2$<br>Experimental *m/z*: 226.1804<br>Theoretical *m/z* of $[M+H]^+$: 226.18016<br>InChI Key: LXKKOLHRNROBTR-GDNZZTSVSA-N<br>SMILES:<br>[H][C@@]1(OC(CC(C)C)=O)C[C@@H]2CC[C@@H](N2C)C1<br>NMR (500 MHz, D_2O) ~3 mg	
**Carbon #<br>(group)**	**^{1}H (ppm)**	**^{13}C (ppm)<br>From HMBC and HSQC**
**1,5** (CH)	3.96 (m, *J* = 5.0 Hz, 2H)	61.05
**2,4** (CH_2)	Axial 1.98 (dd, *J* = 16.6, 5.0 Hz, 2H)<br>Equatorial 2.22 (dt, *J* = 16.6, 4.9 Hz, 2H)	36.64
**3** (CH)<br>- 1 (CO)<br>- 2 (CH_2)<br>- 3 (CH)<br>- 4, 5 (CH_3)	4.92 (t, *J* = 5.0 Hz, 1H)<br>-<br>2.14 (dd, *J* = 7.0, 12.5 Hz, 2H)<br>1.91(m, *J* = 7.0 Hz, 1H)<br>0.78 (d, *J* = 7.0 Hz, 6H)	64.89<br>175.32<br>43.20<br>25.38<br>21.60
**6,7** (CH_2)	1.91 (m, *J* = 7.6 Hz, 2H), 2.12 (m, *J* = 5.0, 7.6 Hz, 2H)	23.40
**8** N(CH_3)	2.58 (s, 3H)	38.44

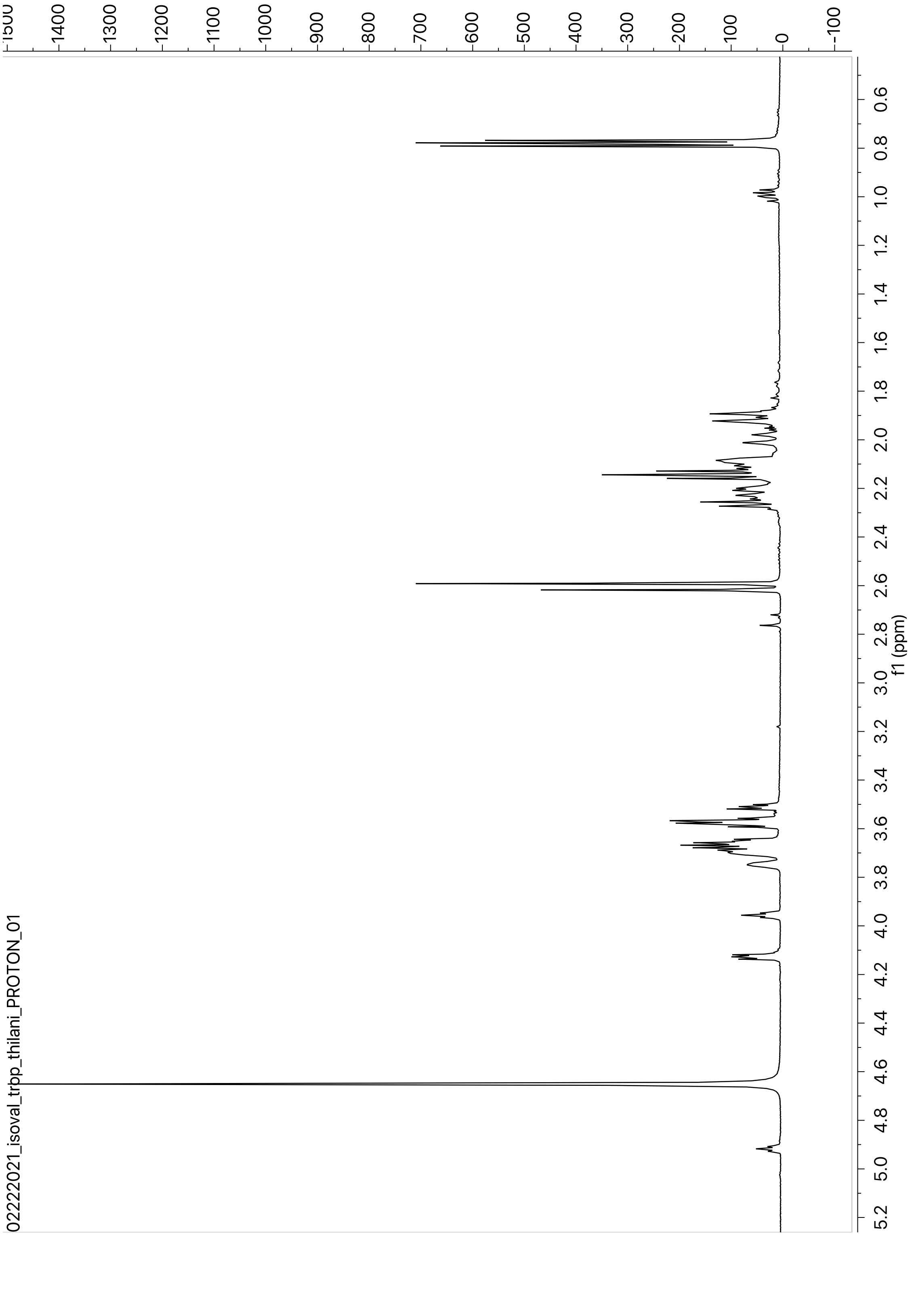

^{1}H NMR spectrum for 3-isovaleryl tropine

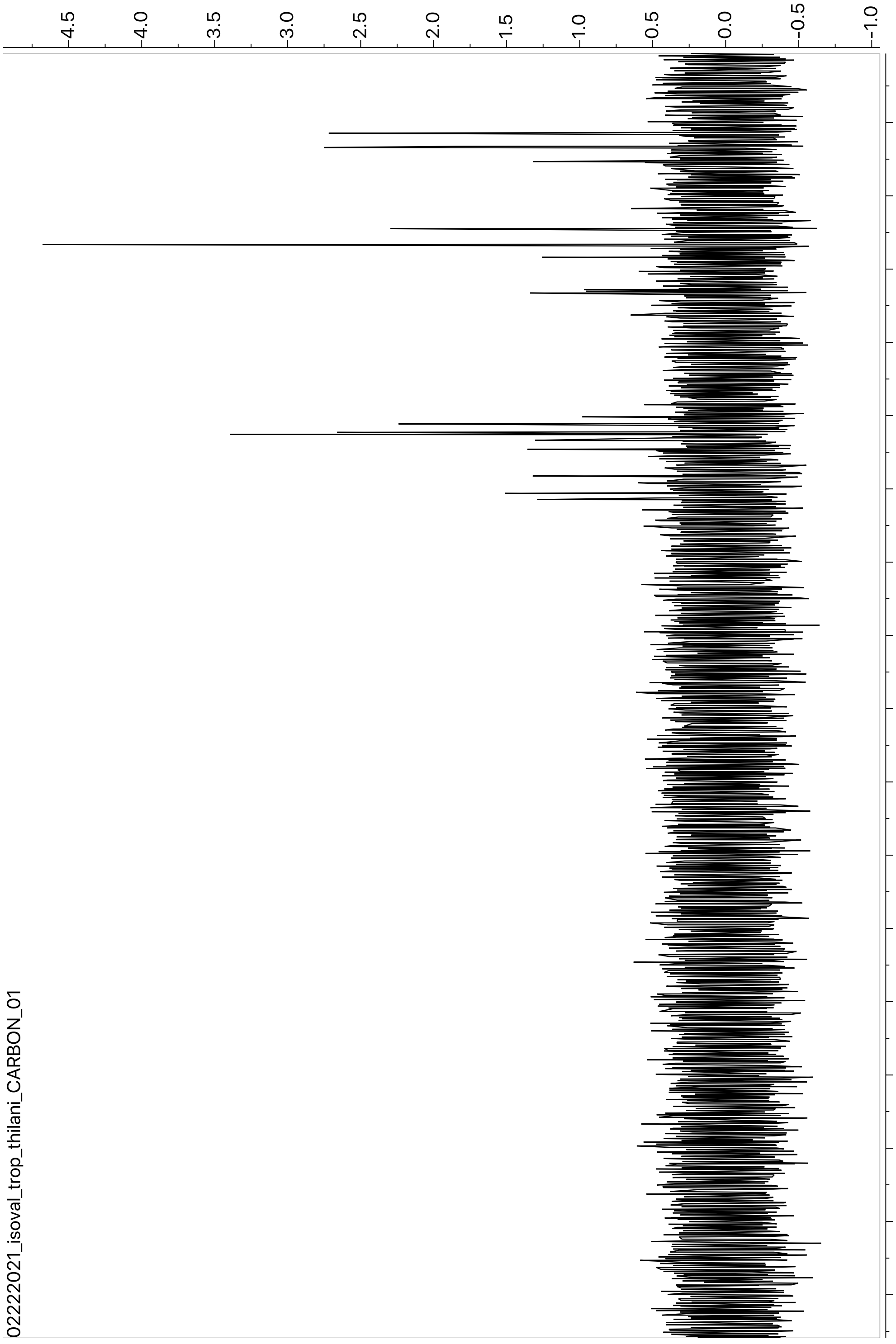

^{13}C NMR spectrum for 3-isovaleryl tropine

K. 3-Senecioyl tropine

NMR chemical shift values for 3-senecioyl tropine

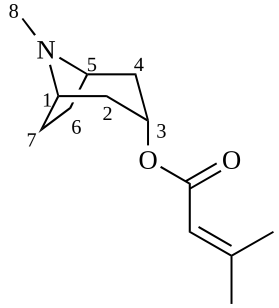	8-methyl-8-azabicyclo[3.2.1]octan-3α-yl 3-methylbut-2-enoate<br>(3-Senecioyl tropine)<br><br>Chemical Formula: $C_{13}H_{21}NO_2$<br>Experimental *m/z* of $[M+H]^+$: 224.1653<br>Theoretical m/z of $[M+H]^+$: 224.16451<br>InChI Key: PFQFRMFXPJMNJJ-GDNZZTSVSA-N<br>SMILES:<br>[H][C@@]1(OC(/C=C(C)\C)=O)C[C@@H]2CC[C@@H](N2C)<br>C1<br>NMR (500 MHz, $CDCl_3$) ~2 mg	
**Carbon #<br>(group)**	**^{1}H (ppm)**	**^{13}C (ppm)**<br>[a] from HMBC
**1,5** (CH)	3.19 (m, *J* = 5.5 Hz, 2H)	60.01
**2,4** (CH_2)	Axial 1.73 (m, *J* = 13.5 Hz, 2H)<br>Equatorial 2.25 (m, *J* = 13.5, 5.5 Hz, 2H)	36.17
**3** (CH)<br>- 1 (CO)<br>- 2 (CH)<br>- 3(C)<br>- 4(CH_3)<br>- 5(CH_3)	5.01 (t, *J* = 5.4 Hz, 1H)<br>-<br>5.64 (hept, *J* = 1.3 Hz, 1H)<br>-<br>2.17 (m, *J* = 2.6, 1.3 Hz, 3H)<br>1.90 (m, *J* = 12.6, 1.3 Hz, 3H)	65.88<br>166.33[a]<br>116.36<br>156.94[a]<br>20.16<br>27.43
**6,7** (CH_2)	2.03 (m, *J* = 5.5, 9.5 Hz, 2H), 2.25 (m, *J* = 5.2 Hz, 2H)	25.53
**8** N(CH_3)	2.33 (s, 3H)	40.06

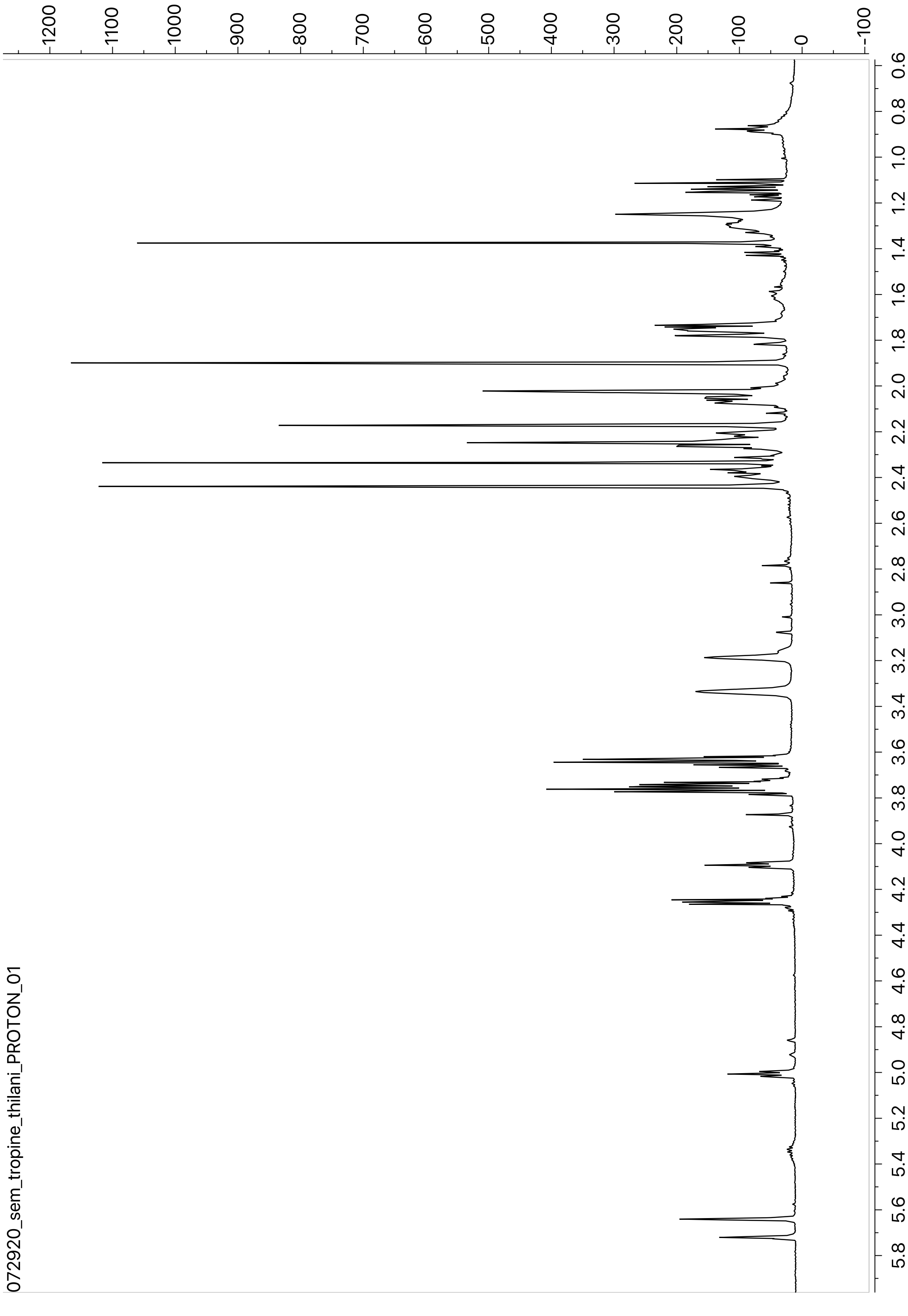

^{1}H NMR spectrum for 3-senecioyl tropine

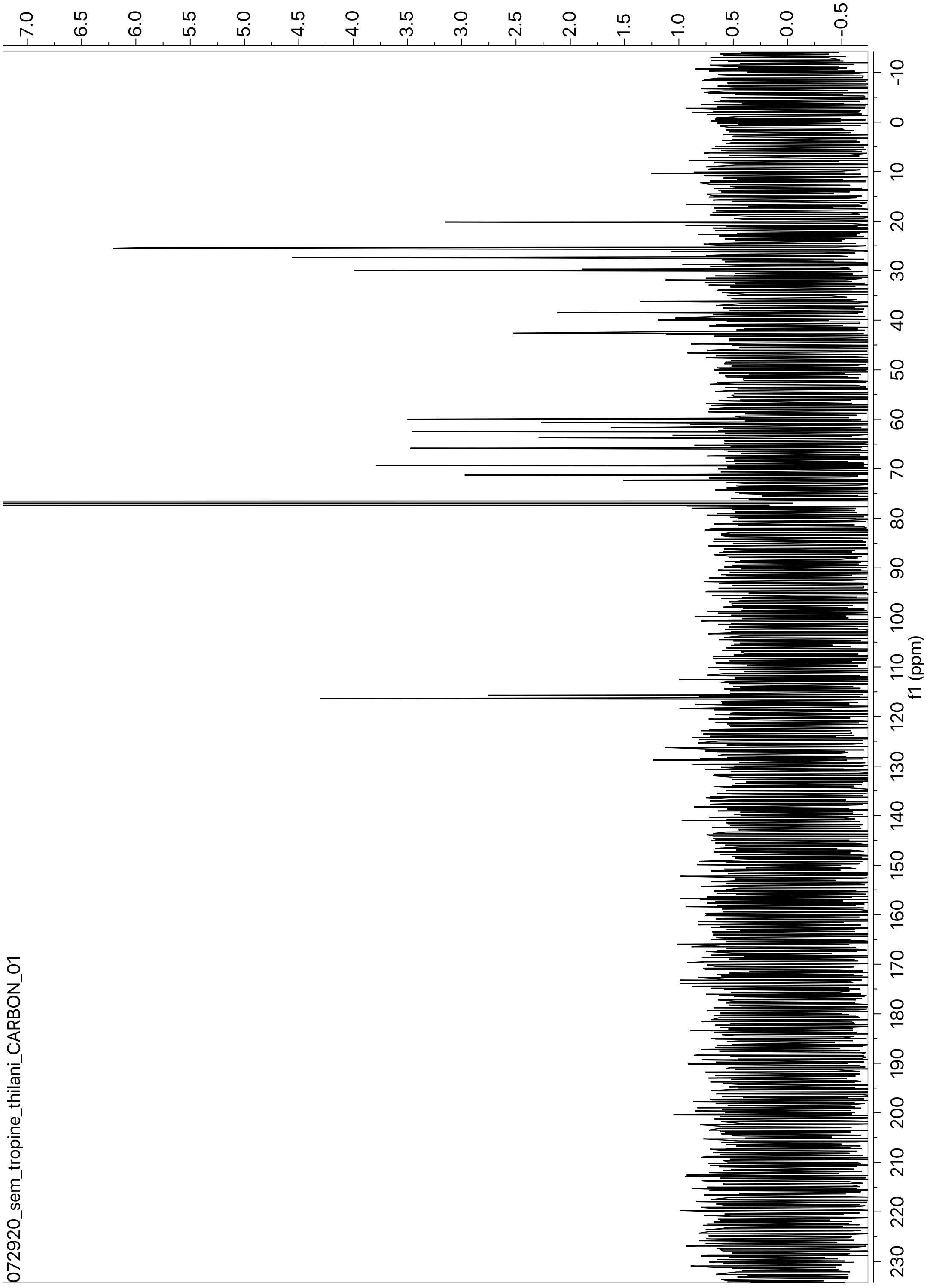

^{13}C NMR spectrum for 3-senecioyl tropine

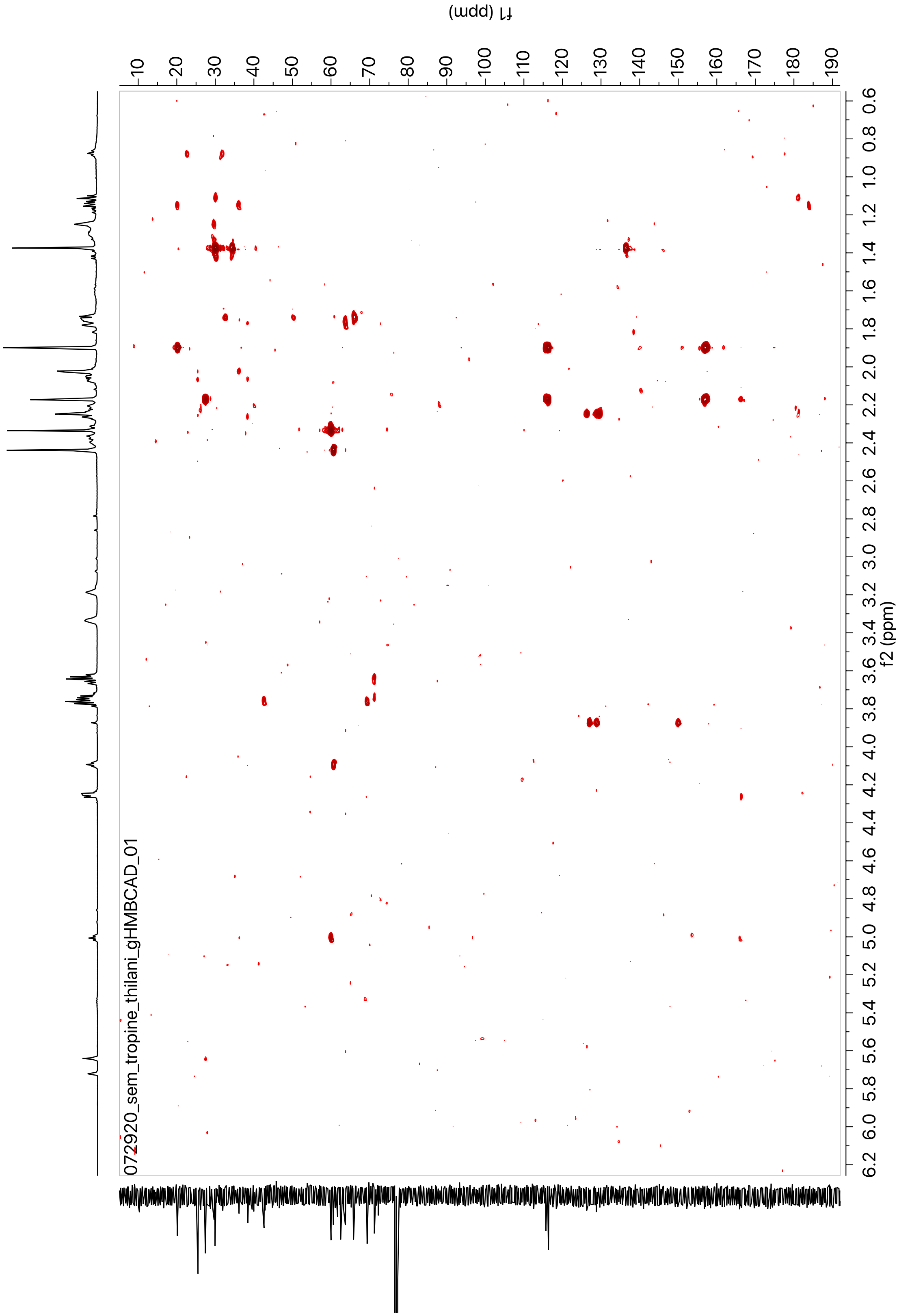

gHMBCAD spectrum for 3-senecioyl tropine

K. 3-Tigloyl tropine

NMR chemical shift values for 3-tigloyl tropine

<table>
<tr><td>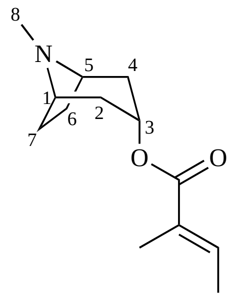</td><td colspan="2">8-methyl-8-azabicyclo[3.2.1]octan-3α-yl (E)-2-methylbut-2-enoate
(3-Tigloyl tropine)

Chemical Formula: $C_{13}H_{21}NO_2$
Experimental m/z: 224.1648
Theoretical m/z of $[M+H]^+$: 224.16451
InChI Key: UVHGSMZRSVGWDJ-LKQNJMEQSA-N
SMILES:
[H][C@@]1(OC(/C(C)=C/C)=O)C[C@@H]2CC[C@@H](N2C)C1
NMR (500 MHz, $CDCl_3$) ~ 1 mg</td></tr>
<tr><th>Carbon #
(group)</th><th>^{1}H (ppm)</th><th>^{13}C (ppm)</th></tr>
<tr><td>1,5 (CH)</td><td>3.44 (m, J = 5.2, 2H)</td><td>61.73</td></tr>
<tr><td>2,4 (CH_2)</td><td>Axial 1.84 (m, J = 13.7, 5.1 Hz, 2H)
Equatorial 2.51 (m, 2H)</td><td>34.60</td></tr>
<tr><td>3 (CH)
- 1 (CO)
- 2 (C)
- 3 (CH_3)
- 4 (CH)
- 5 (CH_3)</td><td>5.12 (t, J = 5.1 Hz, 1H)
-
-
1.84 (d, J = 1.3 Hz, 3H)
6.90 (qq, J = 6.7, 1.3 Hz, 1H)
1.80 (d, J = 6.7 Hz, 3H)</td><td>63.45
169.96
128.86
12.04
140.26
14.39</td></tr>
<tr><td>6,7 (CH_2)</td><td>1.80 (m, J = 5.2, 9.5 Hz, 2H), 2.16 (m, 2H)</td><td>25.25</td></tr>
<tr><td>8 N(CH_3)</td><td>2.52 (s, 3H)</td><td>38.99</td></tr>
</table>

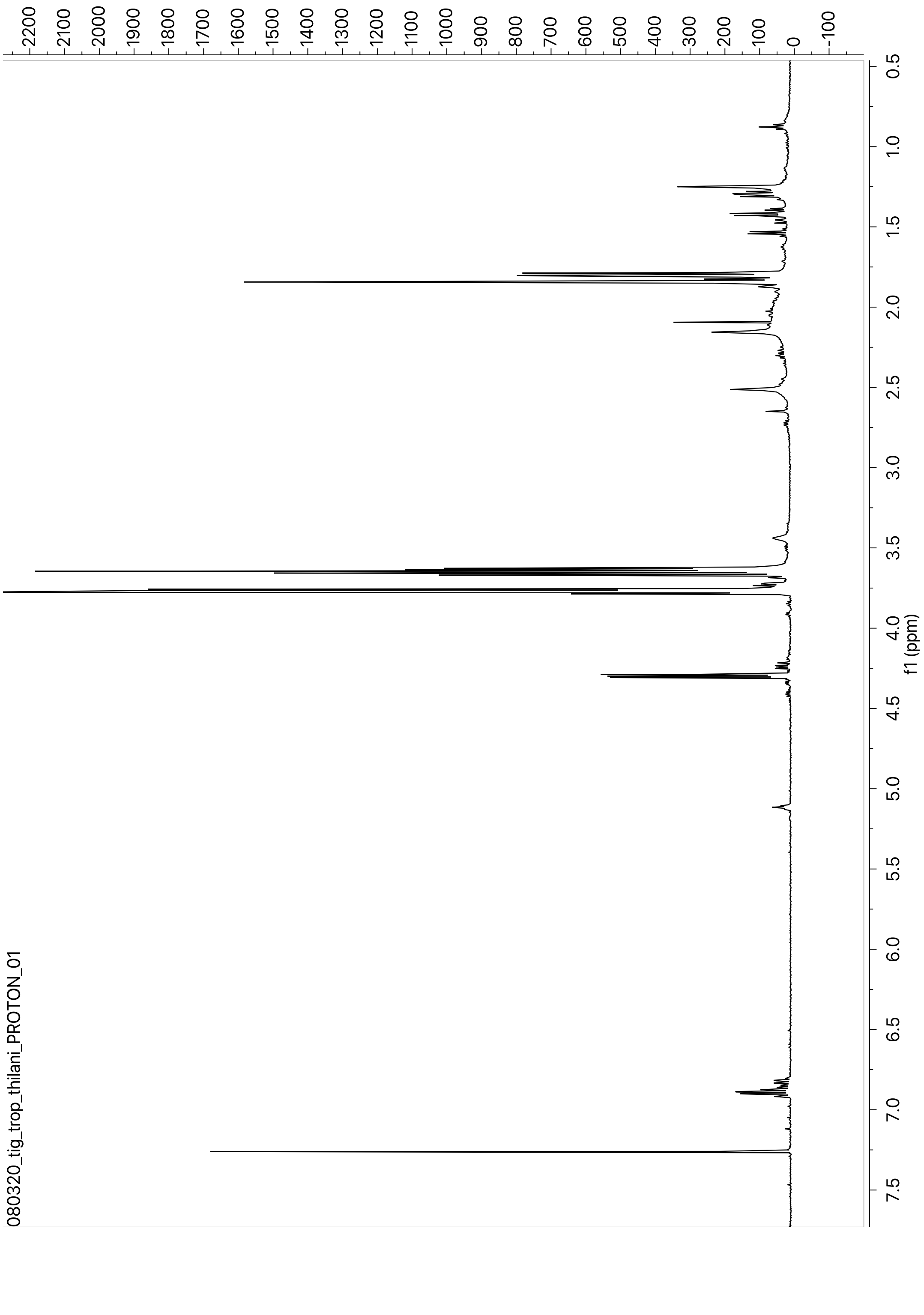

^{1}H NMR spectrum for 3-tigloyl tropine

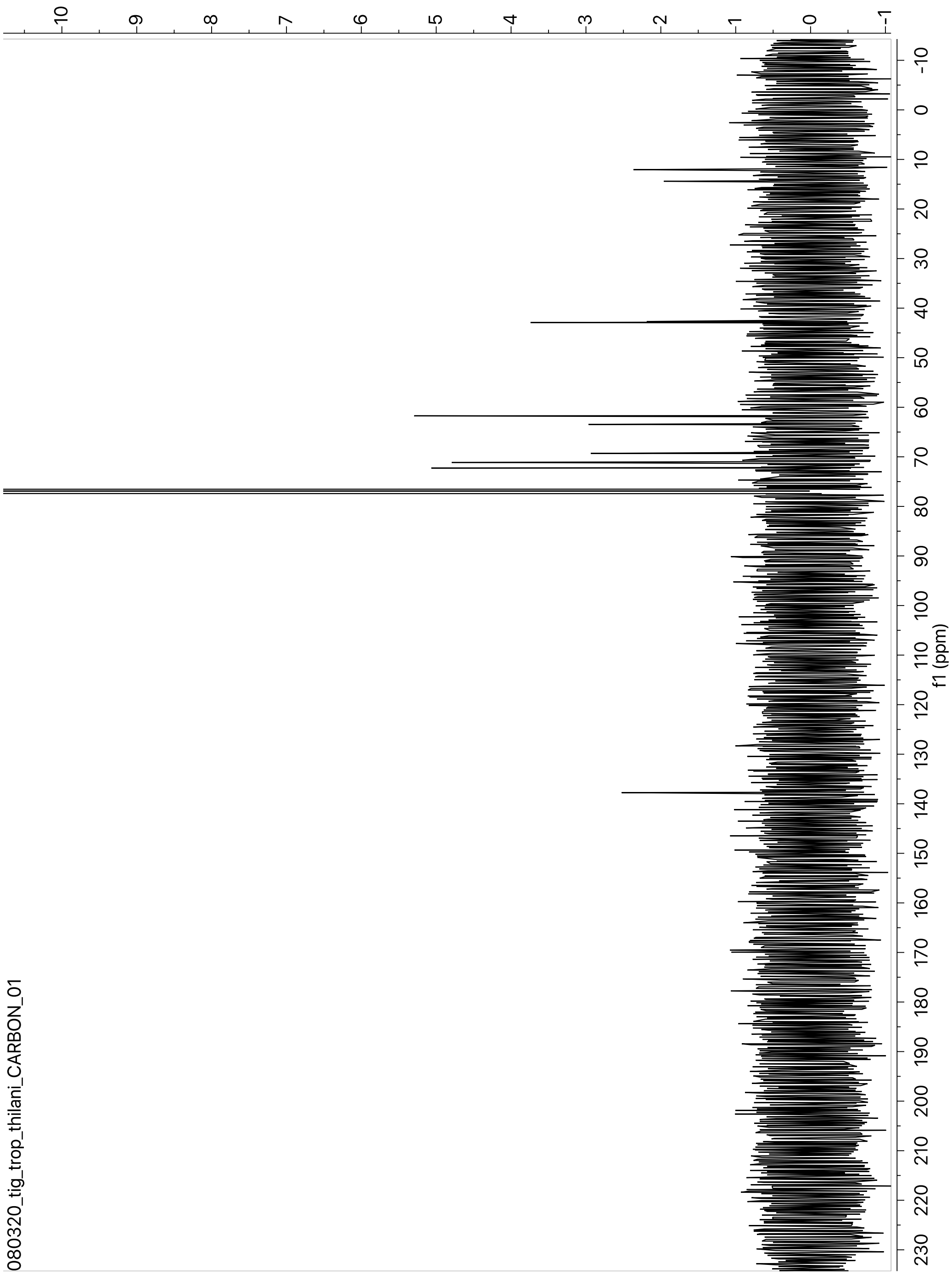

^{13}C NMR spectrum for 3-tigloyl tropine

NORPSEUDOTROPINE ALKALOIDS

L. 3-Tigloyl norpseudotropine

NMR chemical shifts values for 3-tigloyl norpseudotropine

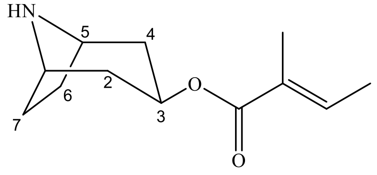

8-azabicyclo[3.2.1]octan-3□-yl (*E*)-2-methylbut-2-enoate
(3-Tigloyl norpseudotropine)

Chemical Formula: $C_{12}H_{19}NO_2$
Experimental m/z $[M+H]^+$: 210.1488
Theoretical m/z $[M+H]^+$: 210.1489
InChI Key: KLFGVBUPYUSQKV-FGUDHKIESA-N
SMILES:
[H][C@]1(OC(/C(C)=C/C)=O)C[C@@H]2CC[C@@H](N2)C1
NMR (500 MHz, D_2O) ~2 mg

**Carbon # (group)**	**^{1}H (ppm)**	**^{13}C (ppm)**
**1,5** (CH)	4.15 (p, *J* = 3.0 Hz, 2H)	54.84
**2,4** (CH_2)	Axial 1.90 (ddd, *J* = 14.2, 11.0, 2.9 Hz, 2H)<br>Equatorial 2.25 (m, 2H)	33.51
**3** (CH)	5.17 (tt, *J* = 11.3, 6.1 Hz, 1H)	65.07
- 1 (CO)	-	169.49
- 2 (C)	-	127.58
- 3 (CH_3)	1.76 (m, 3H)	11.01
- 4 (CH)	6.89 (tdt, *J* = 7.5, 5.7, 2.0 Hz, 1H)	140.19
- 5 (CH_3)	1.76 (m, 3H)	13.69
**6,7** (CH_2)	2.32 (m, 4H)	25.36
**8** N(H)		

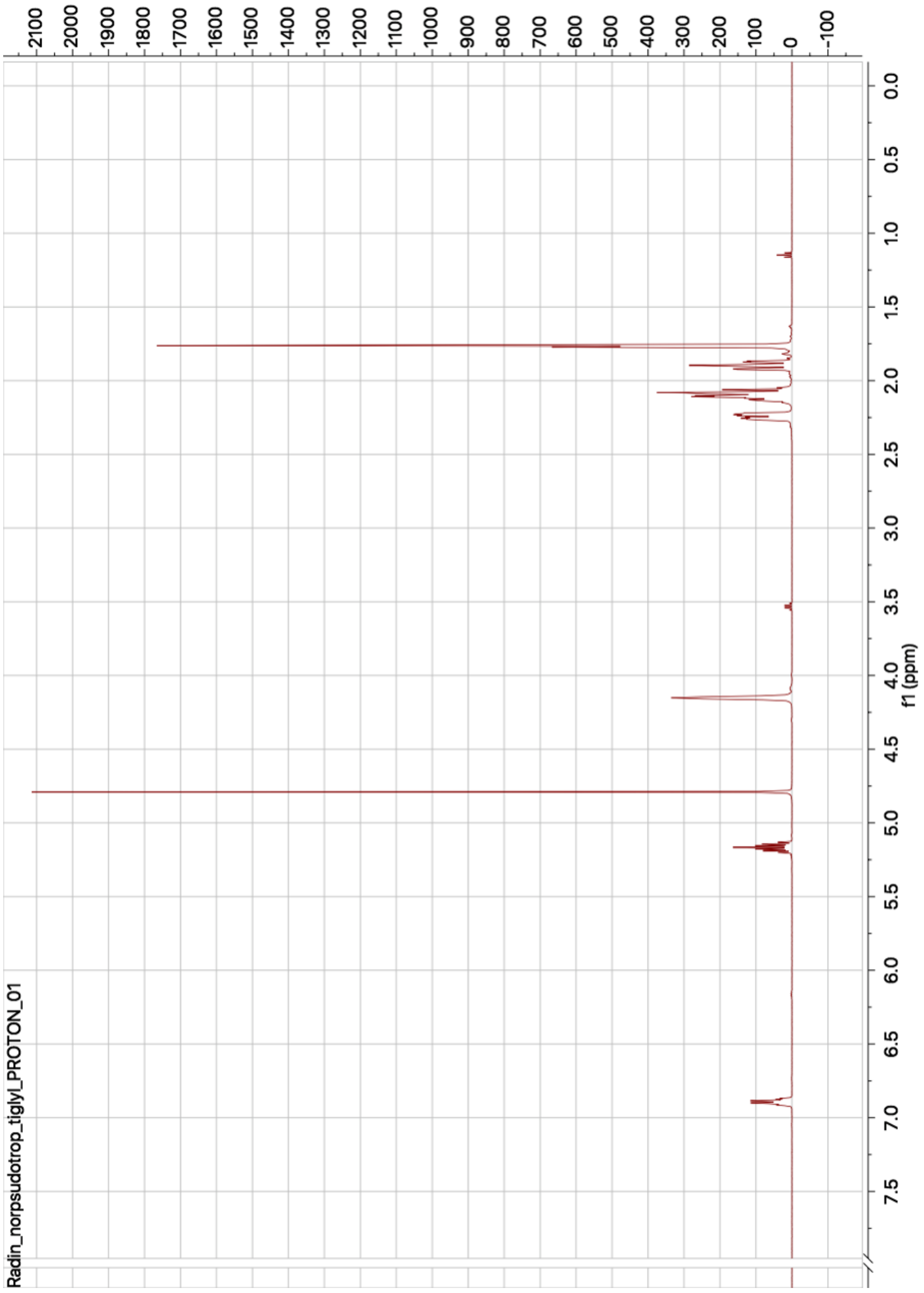

^{1}H NMR spectrum for tigloyl norpseudotropine

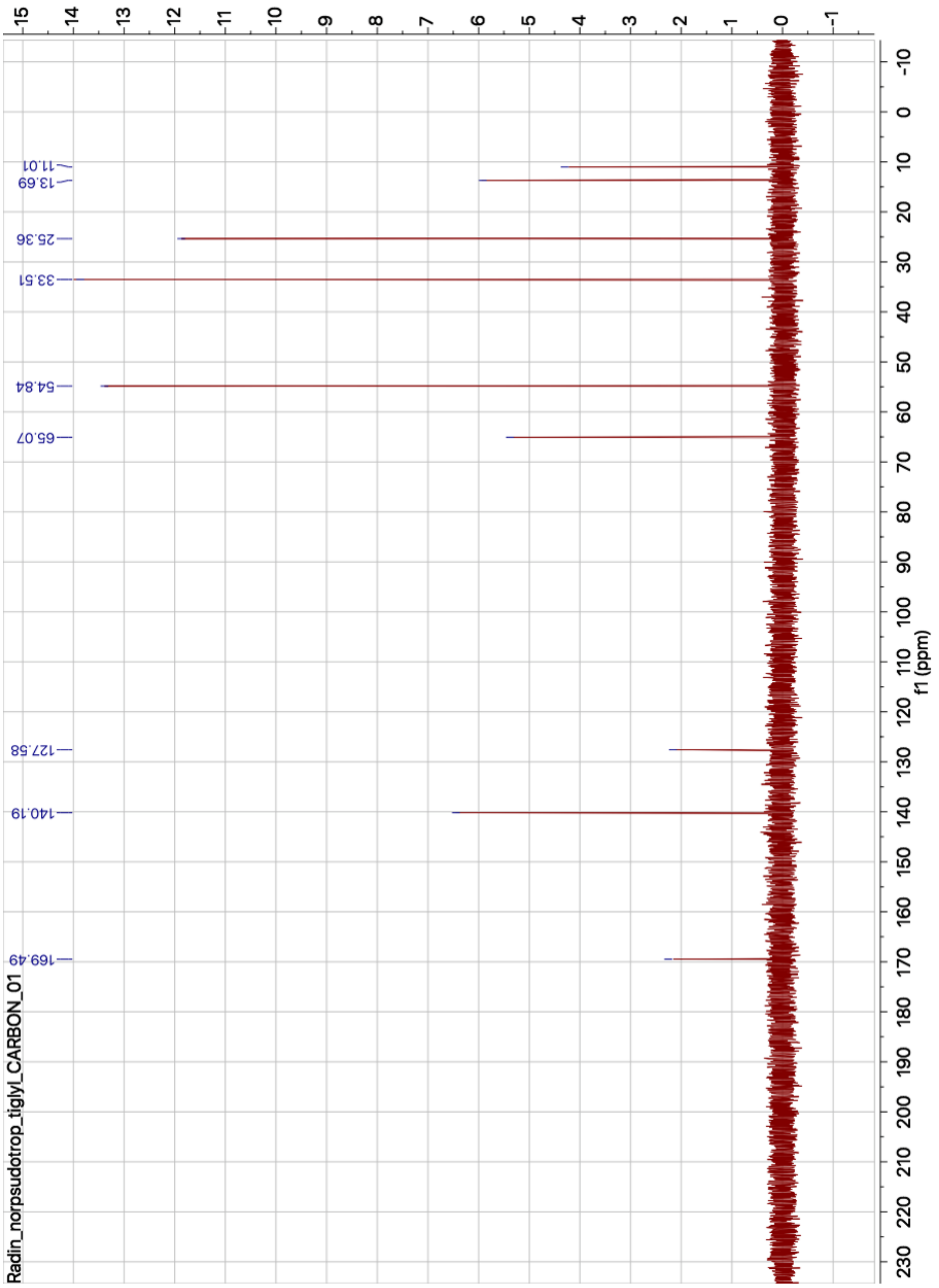

^{13}C NMR spectrum for 3-tigloyl norpseudotropine

M. 3-Tigloyl hydroxynorpseudotropine isomer 1

NMR chemical shifts values for 3-tigloyl hydroxynorpseudotropine isomer 1

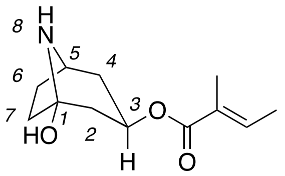	1-Hydroxy-8-azabicyclo[3.2.1]octan-3β-yl (*E*)-2-methylbut-2-enoate<br><br>Chemical Formula: $C_{12}H_{19}NO_3$<br>Experimental *m/z*: 226.1441<br>Theoretical *m/z* of $[M+H]^+$: 226.14377<br><br>InChI key: VJPUKJOSMVWYQS-WOCUHLHXSA-N<br>SMILES:<br>[H][C@]1(OC(/C(C)=C/C)=O)C[C@@]2(O)CC[C@@H](N2)C1<br><br>NMR (600 MHz, $CDCl_3$) ~2 mg	
**Carbon # (group)**	**^{1}H (ppm)**	**^{13}C (ppm)**
**1** C(OH)	-	*a*
**2** (CH_2)	Axial 2.19 (m, *J* =13.2, 11.1 Hz, 1H)<br>Equatorial 2.40 (dd, *J* = 13.1, 6.1 Hz, 1H)	41.90
**3** (CH)<br>- 1 (CO)<br>- 2 (C)<br>- 3 (CH_3)<br>- 4 (CH)<br>- 5 (CH_3)	5.13 (tt, *J* = 11.2, 6.0 Hz, 1H)<br>-<br>-<br>1.79 (m, 3H)<br>6.83 (m, 1H)<br>1.79 (m, 3H)	65.38<br>167.15<br>128.09<br>11.91<br>138.31<br>14.36
**4** (CH_2)	Axial 1.81 (m,*J* = 13.2, 1H)<br>Equatorial 2.14 (m,*J*= 13.1, 3.4,1H)	34.36
**5** (CH)	3.91 (dt, *J* = 7.1, 3.2 Hz, 1H)	51.85
**6** (CH_2)	2.31 (dq, *J* = 13.2, 7.0, 6.4 Hz, 1H), 1.82 (*J*= 13.2, 1H)	25.95
**7** (CH_2)	2.17 (m, 1H), 2.08 (m, 1H)	32.77
**8** N(H)	*b*	-

a. Signal not detected at 25 °C due to rapid tautomerization*
b. Broad signal at 25 °C
*

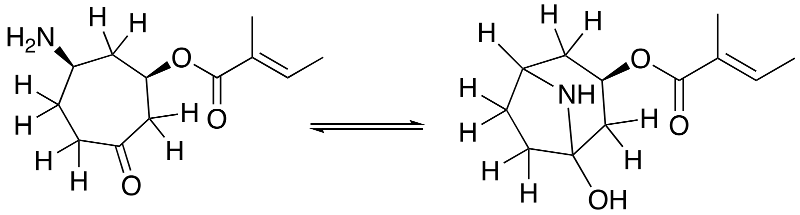

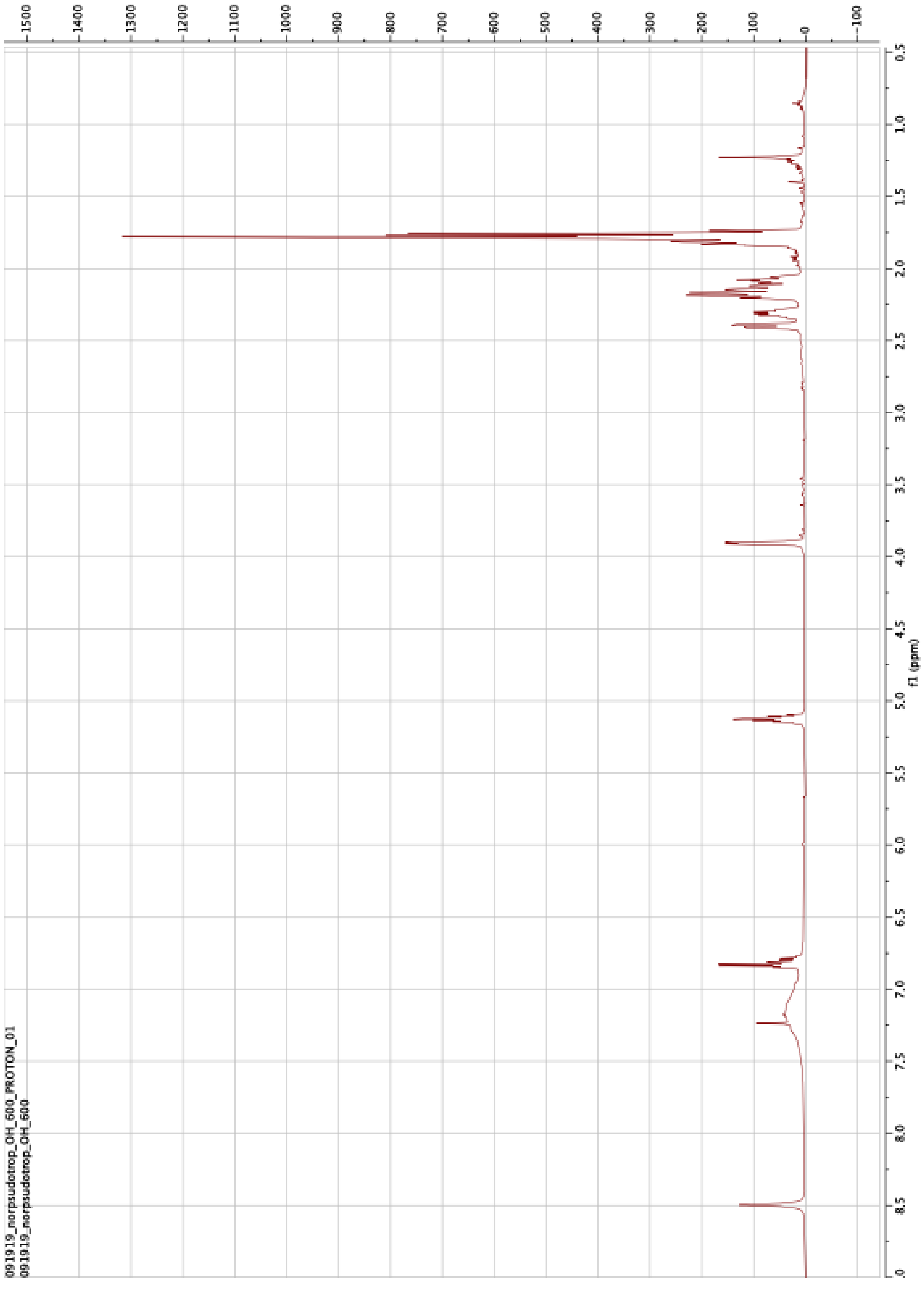

^{1}H NMR spectrum for 3-tigloyl hydroxynorpseudotropine isomer 1

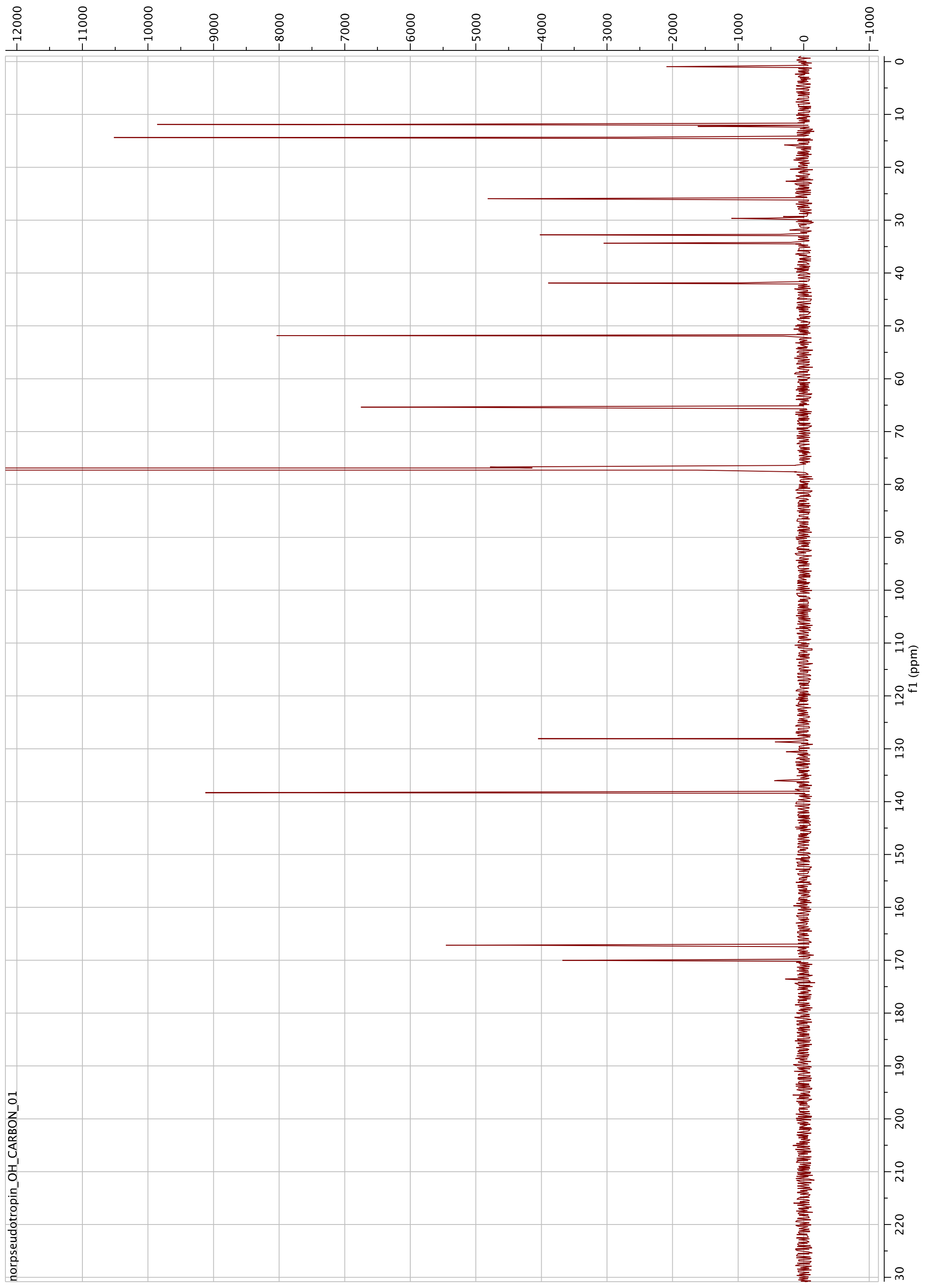

^{13}C NMR spectrum for 3-tigloyl hydroxynorpseudotropine isomer 1

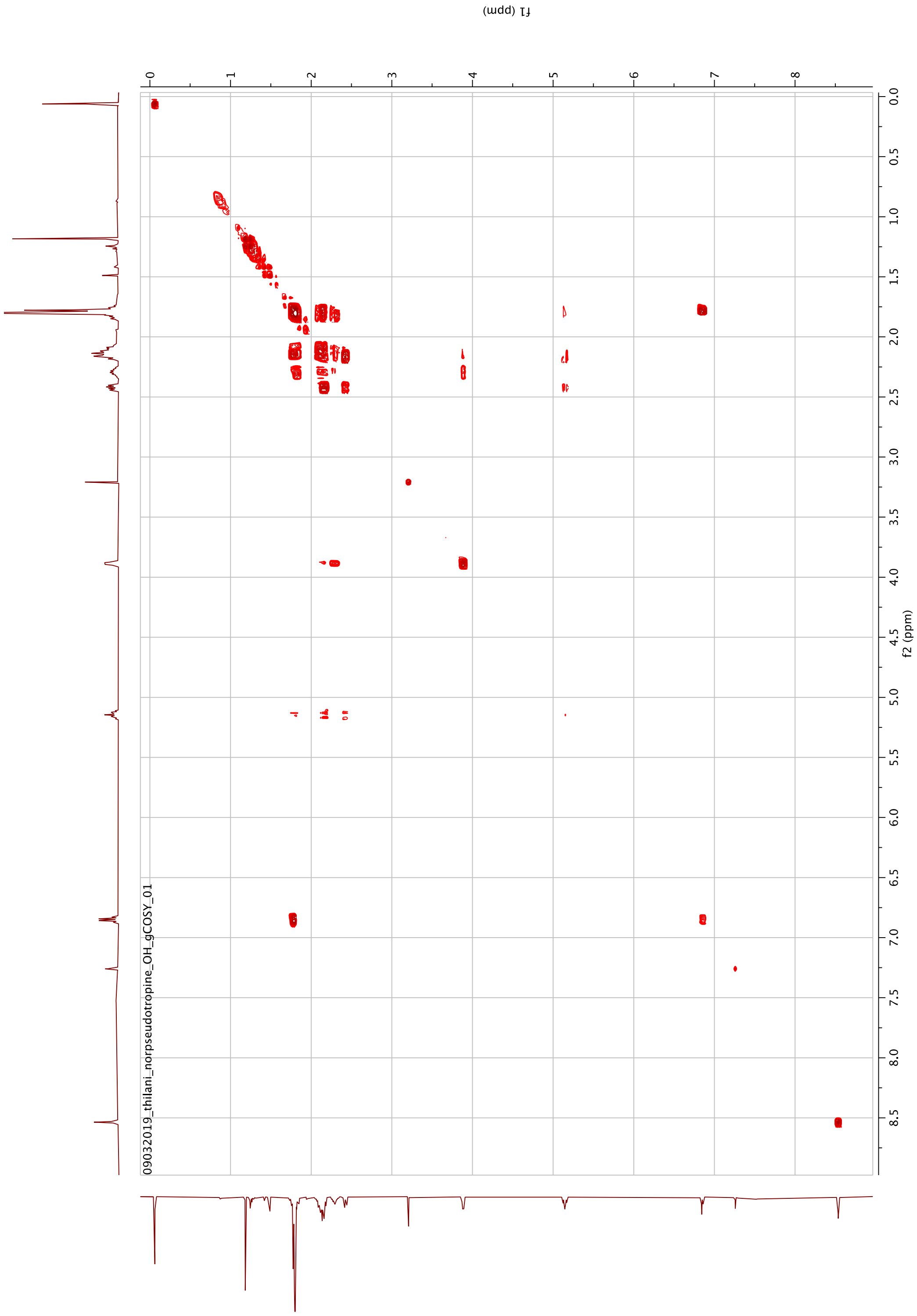

^{1}H-^{1}H gCOSY NMR spectrum for 3-tigloyl hydroxynorpseudotropine isomer 1

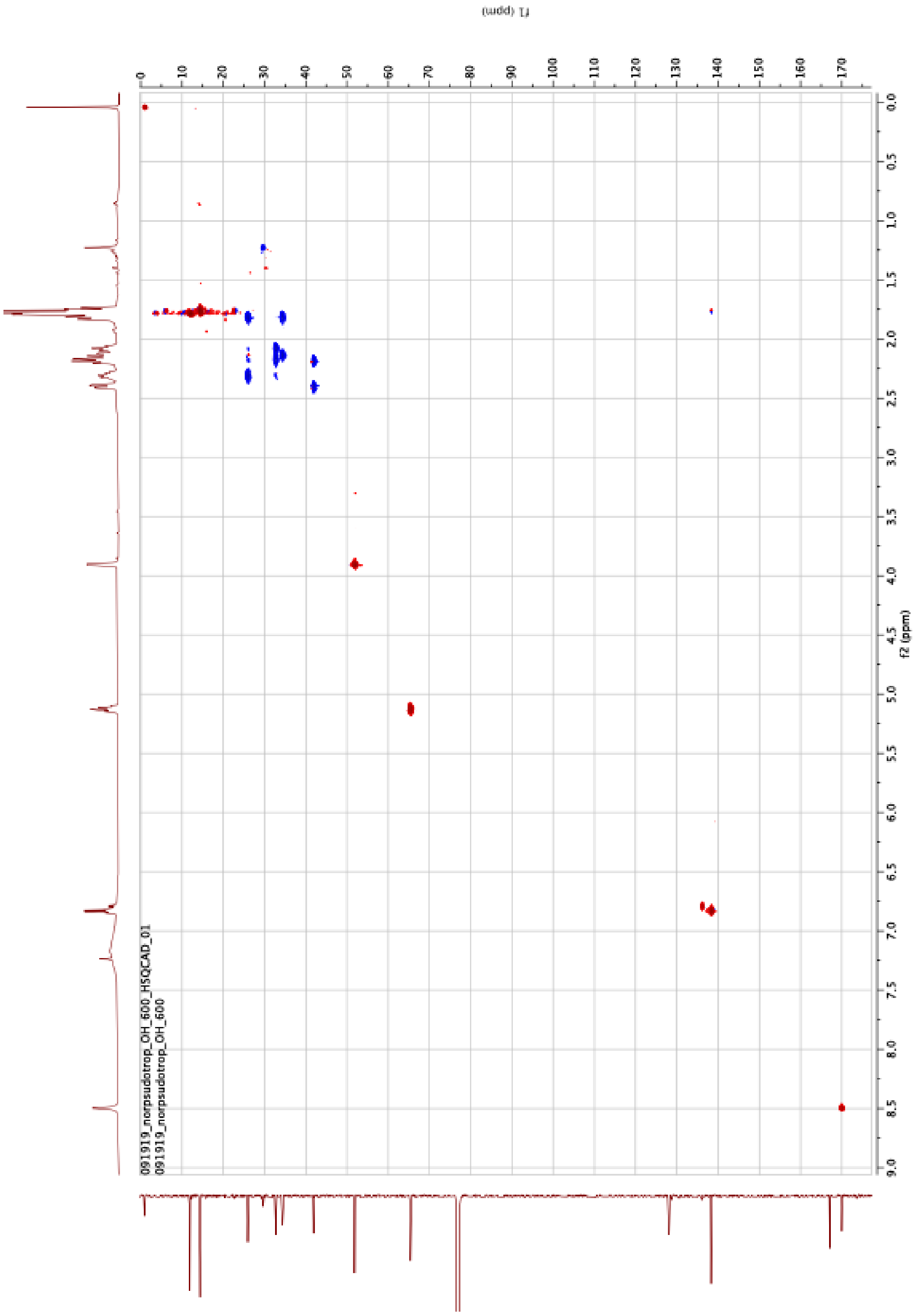

^{1}H-^{13}C gHSQC NMR spectrum for 3-tigloyl hydroxynorpseudotropine isomer 1

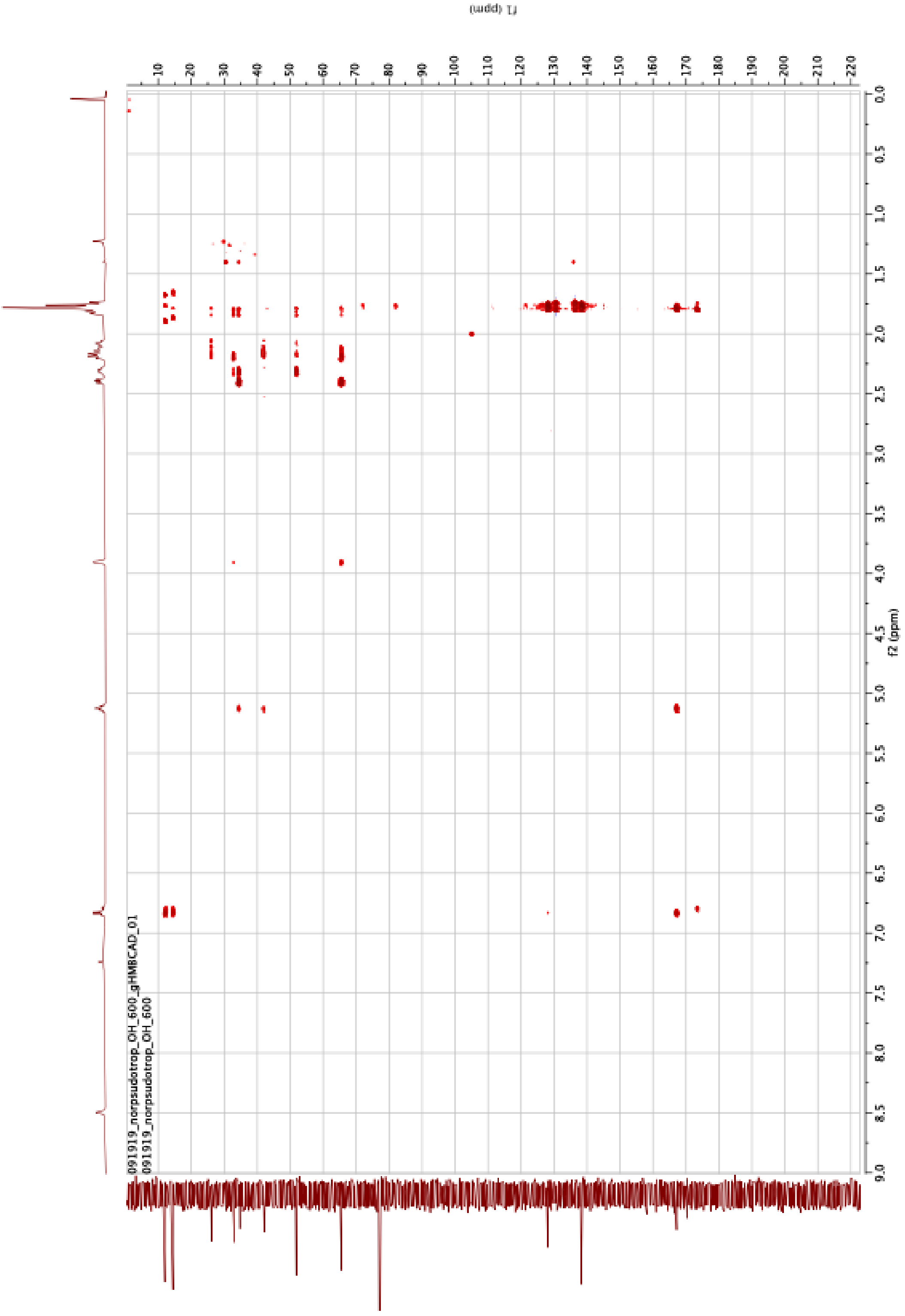

^{1}H-^{13}C gHMBC NMR spectrum for 3-tigloyl hydroxynorpseudotropine isomer 1

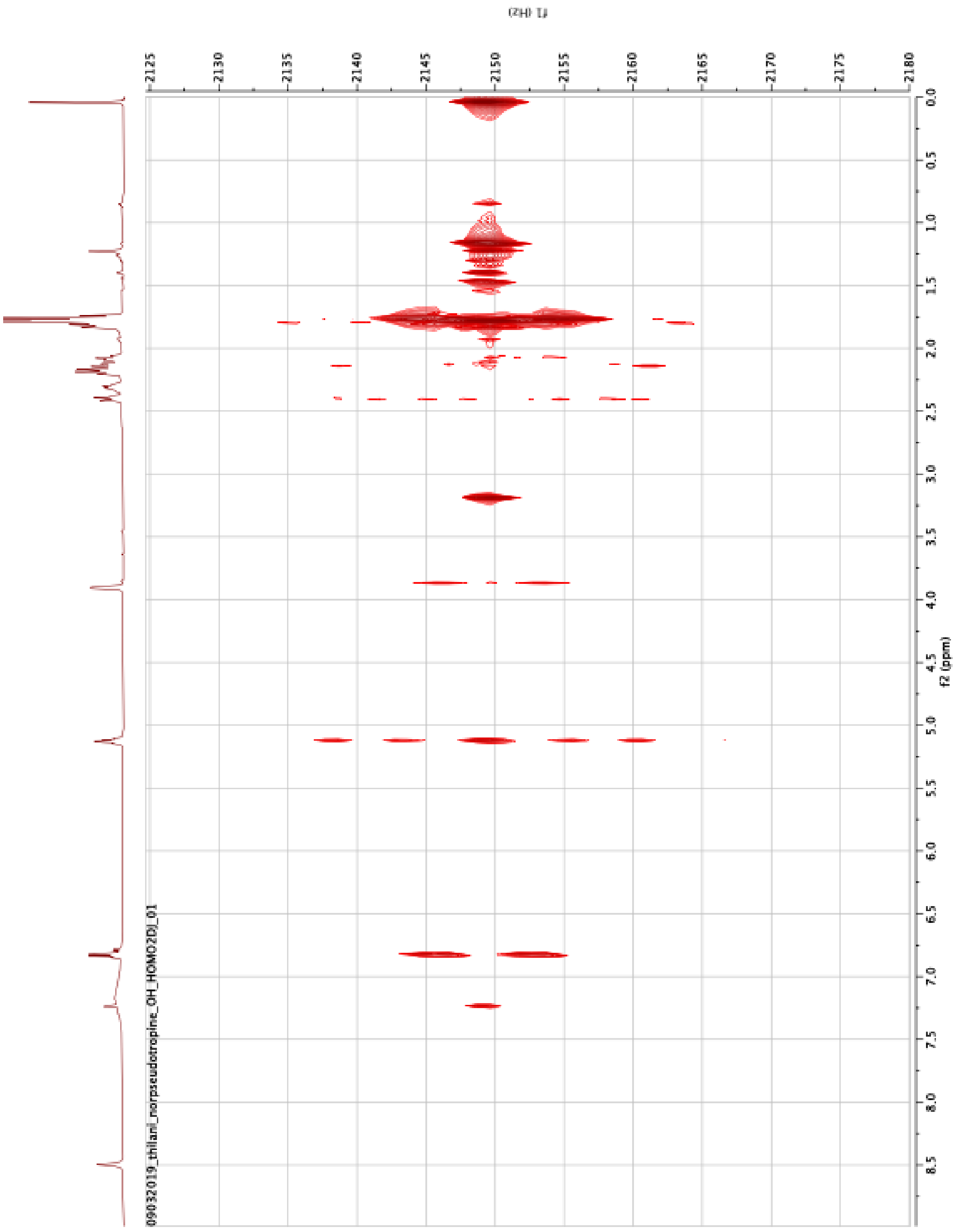

J-resolved ^1H-^1H NMR spectrum for 3-tigloyl hydroxynorpseudotropine isomer 1

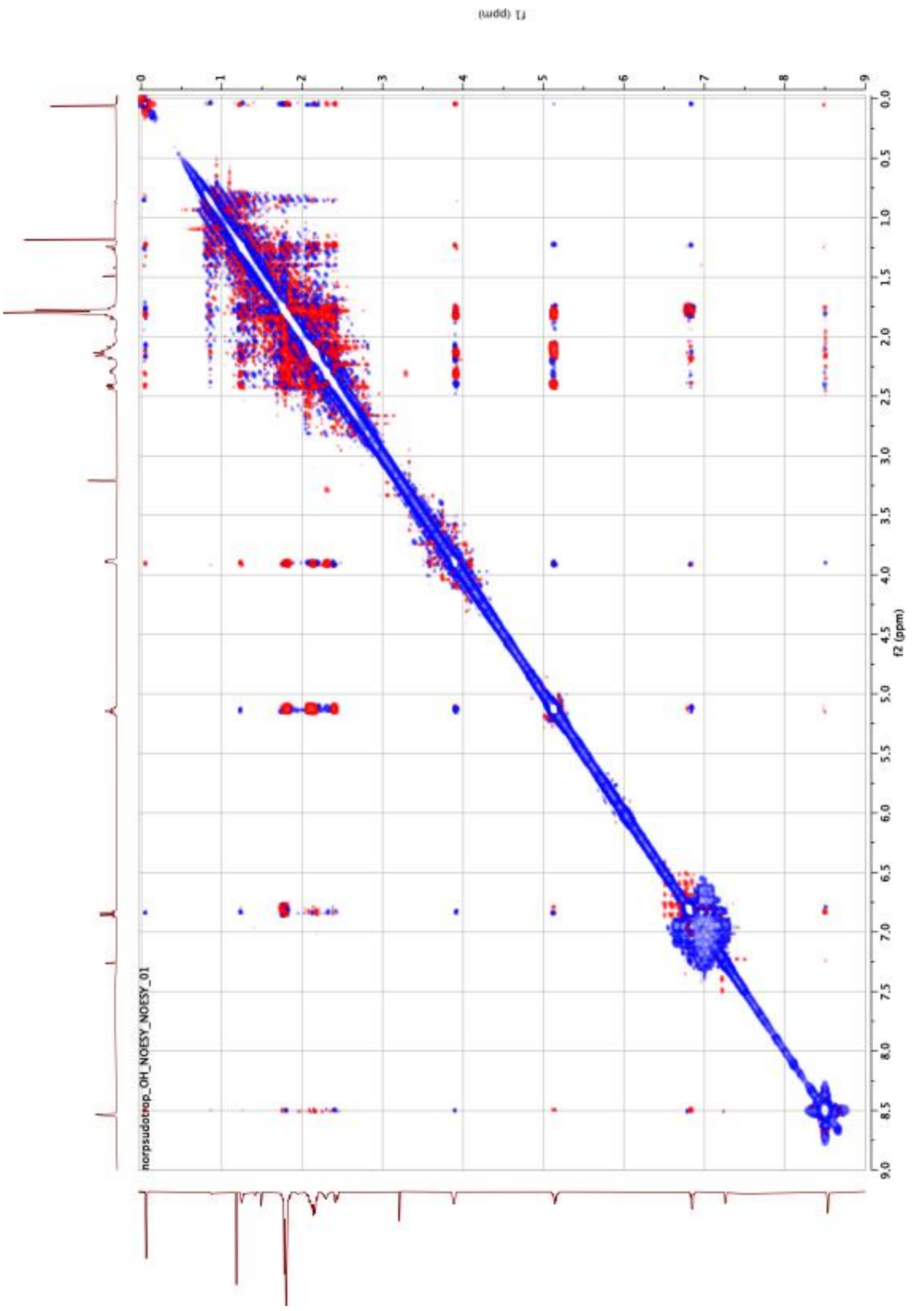

^{1}H-^{1}H NOESY NMR spectrum for 3-tigloyl hydroxynorpseudotropine isomer 1

<u>**N. 3-Tigloyl hydroxynorpseudotropine isomer 2**</u>

NMR chemical shifts values for 3-tigloyl hydroxynorpseudotropine isomer 2

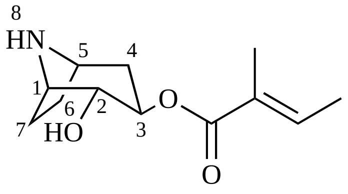	2-Hydroxy-8-azabicyclo[3.2.1]octan-3β-yl (*E*)-2-methylbut-2-enoate<br><br>Chemical Formula: $C_{12}H_{19}NO_3$<br>Experimental *m/z*: 226.1438<br>Theoretical *m/z* of $[M+H]^+$: 226.14377<br>InChI Key: FLUIERQXKMFGPY-WGJUQJDTSA-N<br>SMILES:<br>[H][C@]1(OC(/C(C)=C/C)=O)[C@@](O)([H])[C@@H]2CC[C@@H](N2)C1<br>NMR (600 MHz, $CDCl_3$) ~2 mg

**Carbon # (group)**	**^{1}H (ppm)**	**^{13}C (ppm)**
**1** CH	4.37 (dd, *J* = 7.7, 3.4 Hz, 1H)	72.74
**2** (CH) OH	3.83 (t, *J* = 3.4 Hz, 1H)	63.18
**3** (CH)	4.89 (td, *J* = 11.4, 6.0 Hz, 1H)	64.48
- 1 (CO)	-	167.60
- 2 (C)	-	128.36
- 3 (CH_3)	1.79 (s, 3H)	11.95
- 4 (CH)	6.83 (q, *J*=7.1 Hz, 1H)	138.48
- 5 (CH_3)	1.79 (d, *J*=7.1 Hz, 3H)	14.45
**4** (CH_2)	Axial 1.95 (m, *J* = 11.4, 3.7 Hz, 1H) Equatorial 2.25 (dt, *J* = 6.1, 3.7 Hz, 1H)	32.21
**5** (CH)	4.08 (dd, *J* = 7.0, 3.7 Hz, 1H)	54.30
**6** (CH_2)	2.49 (dd, *J* = 14.8, 7.0 Hz, 1H), 2.16 (m *J* = 3.1, 6.2 Hz, 1H)	39.45
**7** (CH_2)	2.09 (m, J= 7.7, 6.2 Hz, 1H), 1.95 (m, J = 14.8, 3.4 Hz, 1H)	33.76
**8** N(H)	*a*	-

a. Broad signal at 25 °C
2 (CH) OH : H is axial because 1H-2H from H-H J res matches literature value

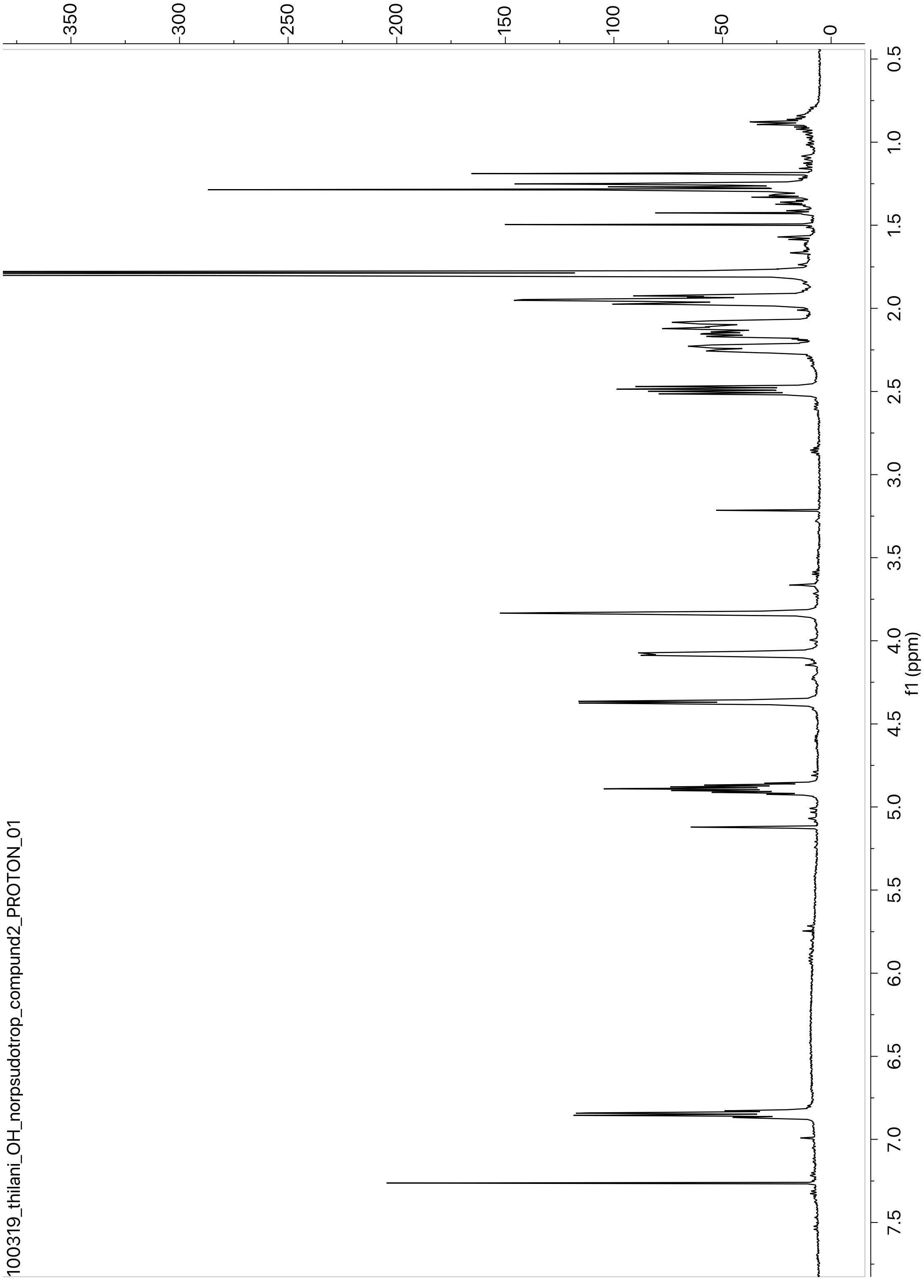

^{1}H NMR spectrum for 3-tigloyl hydroxynorpseudotropine isomer 2

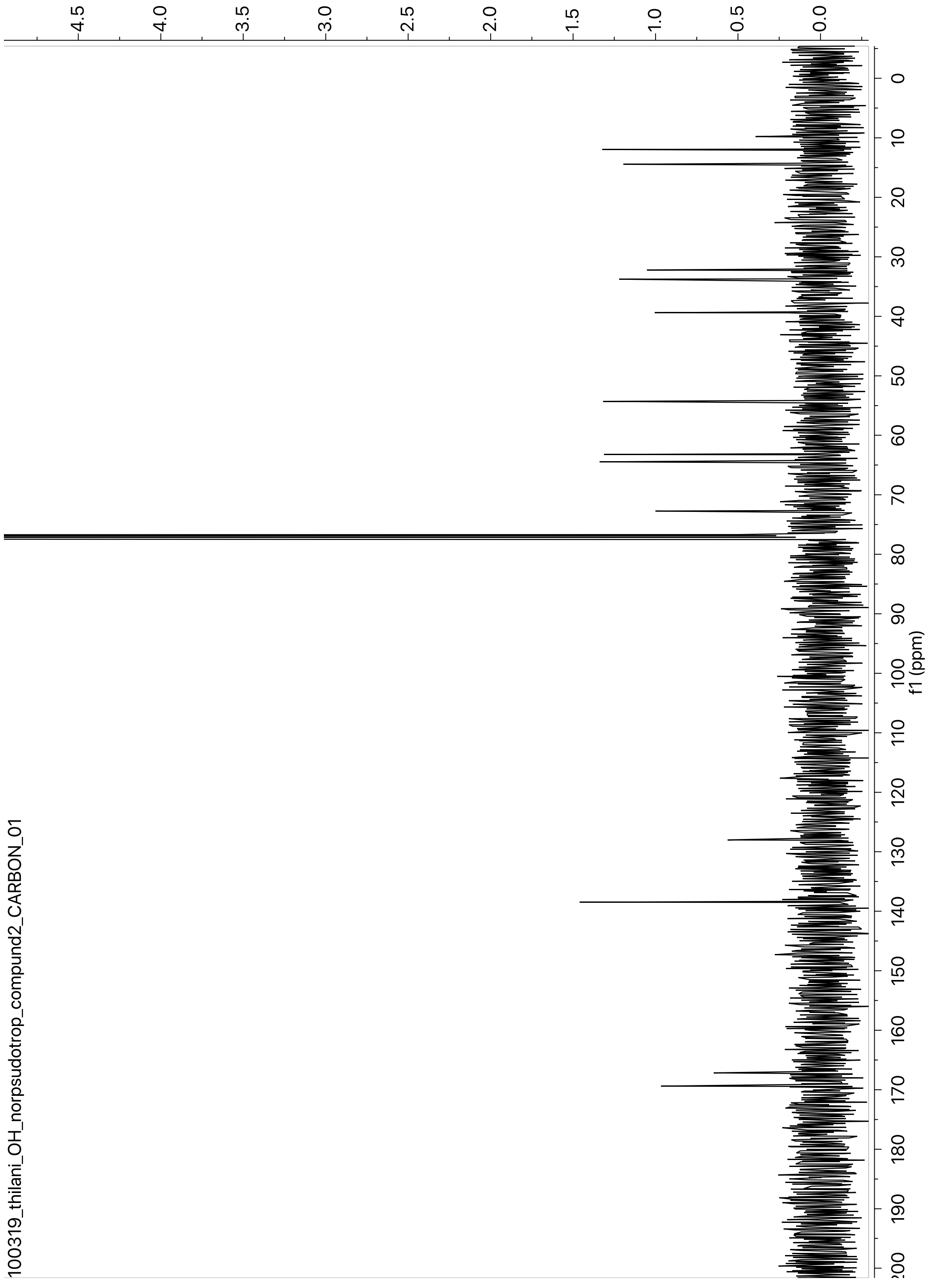

^{13}C NMR spectrum for 3-tigloyl hydroxynorpseudotropine isomer 2
